# Resin-based response of *Pinus pinaster* and *P. radiata* during infection by *Fusarium circinatum*

**DOI:** 10.1093/jxb/erag023

**Published:** 2026-01-20

**Authors:** David Fariña-Flores, Brigida Fernández de Simón, Laura Hernández-Escribano, Lee Robertson, M Teresa Morales Clemente, María Conde, Eugenia Iturritxa, Rosa Raposo

**Affiliations:** Departamento de Ecología y Genética Forestal, Instituto de Ciencias Forestales (ICIFOR-INIA), CSIC, Madrid 28040, Spain; Departamento de Biotecnología-Biología Vegetal, E.T.S. de Ingeniería Agronómica, Alimentaria y de Biosistemas, Universidad Politécnica de Madrid, Madrid 28040, Spain; Departamento de Ecología y Genética Forestal, Instituto de Ciencias Forestales (ICIFOR-INIA), CSIC, Madrid 28040, Spain; Departamento de Ecología y Genética Forestal, Instituto de Ciencias Forestales (ICIFOR-INIA), CSIC, Madrid 28040, Spain; Departamento de Ecología y Genética Forestal, Instituto de Ciencias Forestales (ICIFOR-INIA), CSIC, Madrid 28040, Spain; Departamento de Ecología y Genética Forestal, Instituto de Ciencias Forestales (ICIFOR-INIA), CSIC, Madrid 28040, Spain; Departamento de Ecología y Genética Forestal, Instituto de Ciencias Forestales (ICIFOR-INIA), CSIC, Madrid 28040, Spain; Neiker BRTA, Instituto Vasco de Investigación y Desarrollo Agrario, Arkaute 01192, Spain; Departamento de Ecología y Genética Forestal, Instituto de Ciencias Forestales (ICIFOR-INIA), CSIC, Madrid 28040, Spain; University of Ghent, Belgium

**Keywords:** Defense response, *Fusarium circinatum*, pine pitch canker, *Pinus pinaster*, *Pinus radiata*, resin ducts, terpene-related genes, terpenes, transcriptomic data

## Abstract

Pine pitch canker, caused by the fungus *Fusarium circinatum*, is a disease of economic and ecological importance worldwide. In Spain, *Pinus radiata* and *P. pinaster* are the two species most affected by the pathogen. *Pinus radiata* is one of the most susceptible species to the disease, while *P. pinaster* is moderately resistant. As part of the defensive response to invading organisms, pines contain and *de novo* synthesize resin, mainly composed of terpenes. Resin is accumulated and produced in constitutive and induced resin ducts. We compared the anatomical resin system and terpene profiles in both species infected with *F. circinatum* to better understand the resin-based defense mechanisms against this pathogen. We also used a previous transcriptomic study of the interaction between *P. pinaster* and *F. circinatum* to analyse the expression of terpene-related genes. This study shows that the *F. circinatum*-susceptible species *P. radiata* induced larger resin ducts and produced more resin than the moderately resistant *P. pinaster*, a result similar to that observed with other pathogens that use resin ducts to colonize the host. By comparing the terpene profiles of both species, we identified some terpenes that may contribute to the differential resistance between species. This study provides comprehensive information on terpene content and profiles.

## Teaser

*Fusarium circinatum* fungal infection of susceptible *Pinus radiata* induced larger resin ducts producing more resin, with a different terpene profile, than in the moderately resistant *P. pinaster*.

## Introduction

Conifers produce large quantities of oleoresin (hereafter ‘resin’) in their tissues (Phillips and Croteau, 1999), which is stored in specific anatomical structures (Phillips and Croteau, 1999; Hudgins *et al*., 2004; Celedon and Bohlmann, 2019). Resin is mainly composed of terpenoids (also known as terpenes), which include monoterpenes (MTs), sesquiterpenes (STs), neutral diterpenes (DTs), and diterpene resin acids (DRAs) (Phillips and Croteau, 1999; Celedon and Bohlmann, 2019; Kopaczyk *et al*., 2020). Resin has a defensive function against herbivores and pathogens (Franceschi *et al*., 2005; Keeling and Bohlmann, 2006; Mumm and Hilker, 2006; Eyles *et al*., 2010; Celedon and Bohlmann, 2019; Kopaczyk *et al*., 2020). It contributes to sealing wounds and forming a physical barrier that prevents the entrance of invaders (Keeling and Bohlmann, 2006; Eyles *et al*., 2010; Celedon and Bohlmann, 2019; Kopaczyk *et al*., 2020), and it acts as a chemical defense containing toxic or inhibitory compounds against pathogens and herbivores (Phillips and Croteau, 1999; Eyles *et al*., 2010; Krokene and Nagy, 2012; Fraser *et al*., 2016; Celedon and Bohlmann, 2019).

Among conifers, pines have an interconnected system of axial and radial ducts distributed throughout the plant, where constitutive resin is abundantly produced (Phillips and Croteau, 1999), accumulated, and translocated (Franceschi *et al*., 2005; Vázquez-González *et al*., 2019). Under wounding or infection, pines respond by inducing the formation of new resin ducts, known as traumatic resin ducts (TRDs), which form mainly in the xylem but also in the cortex (Franceschi *et al*., 2005; Kolosova and Bohlmann, 2012). Resin production and accumulation take place in both constitutive and induced ducts (Franceschi *et al*., 2005; Krokene and Nagy, 2012). The induction of TRDs leads to increased resin biosynthesis and accumulation with enhanced resin flow (Franceschi *et al*., 2005). Changes in the chemical terpene composition of the induced resin are produced by enhancing or inhibiting terpene biosynthesis (Keeling and Bohlmann, 2006; Celedon and Bohlmann, 2019; Kopaczyk *et al*., 2020).

In general, the amount of resin that pines exude in response to biotic stresses is positively linked with tree resistance (Nagy *et al*., 2006; Krokene and Nagy, 2012). Several studies have associated constitutive and induced resin duct traits with resistance to different pathogens and herbivores (Kane and Kolb, 2010; Ferrenberg *et al*., 2014; Vázquez-González *et al*., 2020). Increases in the size, abundance, and density of resin ducts are traits that are frequently associated with resistant trees, being important for both defensive chemistry and more efficient physical defenses (Nagy *et al*., 2006; Krokene and Nagy, 2012). As part of the plant defensive system, resin chemistry and duct characteristics are adaptive traits resulting from an evolutionary process in the interaction of trees with invading organisms (Keeling and Bohlmann, 2006; Krokene and Nagy, 2012). Conifers successfully defend themselves against most invaders, but some have evolved different strategies that overcome tree defenses or use terpenes for their own benefit (Keeling and Bohlmann, 2006; Celedon and Bohlmann, 2019; Vázquez-González *et al*., 2020).

As part of the induced defense response to pests and pathogens, the *de novo* formation of TRDs in the xylem is accompanied by an increase in resin biosynthesis (Nagy *et al*., 2000; Celedon and Bohlmann, 2019). Different studies have compared resin duct traits in the context of host susceptibility to pathogens. The results suggest that the ability of pathogens that use resin canals to spread within plants is favored by larger duct size, which contradicts expectations (Krokene and Nagy, 2012; Martín-Rodrigues *et al*., 2013; Rodríguez-García *et al*., 2023). For example, pines highly susceptible to the nematode *Bursaphelenchus xylophilus* Steiner & Buhrer showed wider constitutive canals in the cortex and smaller canals in the xylem than did less susceptible species, and induced xylem canals were more frequent in susceptible pines (Rodríguez-García *et al*., 2023). *Fusarium circinatum* Nirenberg & O’Donnell is a pathogen that uses resin ducts to spread within the host (Martín-Rodrigues *et al*., 2013), but the influence of duct size on pathogen resistance has not been studied. The exogenous application of methyl jasmonate reveals different strategies for altering the resin duct structure of pines (López-Villamor *et al*., 2021). *Pinus sylvestris* L. and *P. radiata* D.Don, but not *P. pinaster* Ait. or *P. pinea* L., increased the number, density, and mean size of resin ducts in the secondary xylem but not in the cortex.

*Fusarium circinatum* is the causal agent of pine pitch canker (PPC) disease. In Europe, the pathogen has become established on the Atlantic coast of the Iberian Peninsula, where the most productive pine plantations of *P. pinaster* and *P. radiata* are found. The susceptibility of pine species to PPC disease varies widely (Martín-García *et al*., 2018). The most susceptible species is *P. radiata* (Gordon *et al*., 1998; Hodge and Dvorak, 2000), which is an exotic species in Spain, while *P. pinaster* is moderately resistant (Elvira-Recuenco *et al*., 2014). In general, native species grown in Spain show low susceptibility to PPC disease (Iturritxa *et al*., 2012). The characteristic symptoms of PPC disease are sunken cankers on tree stems and branches with abundant resin. Needles become necrotic above the infection point, causing branch dieback due to the obstruction of water flow by girdling cankers (Wingfield *et al*., 2008). When branch infections are extensive in a tree, they cause canopy dieback and tree mortality (Drenkhan *et al*., 2020). PPC disease is distributed worldwide (Drenkhan *et al*., 2020) and is considered a major threat to pines, damaging tree growth and wood quality (Wingfield *et al*., 2008; Martín-García *et al*., 2019).

Qualitative and quantitative changes in conifer terpenes have been studied in response to invading organisms (e.g. Zamponi *et al*., 2007; Wallis *et al*., 2008; Liu *et al*., 2021; Trujillo-Moya *et al*., 2022; Ghosh *et al*., 2024). Specifically for *P. pinaster* and *P. radiata* species, changes in terpenes have been previously studied (Moreira *et al*., 2013; Lombardero *et al*., 2019; Lundborg *et al*., 2019) in response to feeding by different insects (*Hylobius abietis* L. and *Thaumetopoea pityocampa* Denis & Schiffermüller), or following the application of methyl jasmonate. Reports have shown that the response in plant tissues (needles and stems) is specific to the tissue type and the herbivores species causing damage (Moreira *et al*., 2013). A recent study reported changes in some terpenes in response to *F. circinatum*, its vector *Tomicus piniperda* L., and both organisms together (Lombardero *et al*., 2019). The results showed that *P. pinaster* increased terpenes following attack by *T. piniperda* but did not respond to *F. circinatum*, while *P. radiata* increased terpene concentrations in response to either *F. circinatum* or *T. piniperda*. This study addressed the effect of a limited number of terpenes from the total profile, mainly MTs, and did not include DRAs. DRAs have received less attention than the other resin groups despite some showing strong antifungal properties (Kusumoto *et al*., 2014), but both pine species strongly increased DRA concentrations in the stems after *Hylobius abietis* feeding, and the response was greater in *P. pinaster* than in *P. radiata* (López-Goldar *et al*., 2020).

Terpenes in conifers are synthesized via two metabolic pathways: the mevalonate (MVA) pathway, which is located in the cytosol, and the methyl-erythritol phosphate (MEP) pathway, which is located in the chloroplast (Celedon and Bohlmann, 2019; Alicandri *et al*., 2020). From these two routes, isopentenyl diphosphate and dimethylallyl diphosphate [two five-carbon molecules (C_5_)] are obtained and assembled, producing geranyl diphosphate (C_10_), farnesyl diphosphate (C_15_), and geranylgeranyl diphosphate (C_20_) (Schmidt *et al*., 2011; Pazouki and Niinemets, 2016; Alicandri *et al*., 2020). These compounds are the precursors of MTs, STs, and DTs, respectively (Bohlmann *et al*., 1998), and are converted into a high number of terpenes by the action of terpene synthases (Keeling and Bohlmann, 2006; Pazouki and Niinemets, 2016; Celedon and Bohlmann, 2019; Alicandri *et al*., 2020). In recent years, progress has been made in describing the biochemical functionality of terpene synthases (reviewed in Celedon and Bohlmann, 2019), which explains the diversity of constitutive and induced terpenes. Similarly, the annotation of the conifer genome and transcriptome has allowed advances in the identification of terpene synthase genes (Schmidt *et al*., 2011; Warren *et al*., 2015; Celedon and Bohlmann, 2019).

Different studies on the interaction of *Pinus* spp. under *F. circinatum* challenge have been carried out using a dual RNA sequencing approach. These studies have emphasized the role of phytohormone regulation in species resistance. Transcriptomic analyses of hormone regulation indicated an earlier response to *F. circinatum* infection in *Pinus tecunumanii* Eguiluz & J.P.Perry, which is more resistant than *Pinus patula* Schiede ex Schltdl. y Cham. (Visser *et al*., 2019), and in *P. pinea*, which is more resistant than *P. radiata* (Zamora-Ballesteros *et al*., 2021). In the case of *P. pinaster*, transcriptomic analysis at 3, 5, and 10 days post inoculation (dpi) revealed that the moderate resistance to *F. circinatum* could be explained by the activation of phytohormone signaling pathways as early as 3 dpi, involving cross-talk between salicylic acid, jasmonic acid, ethylene, and possibly auxins (Hernandez-Escribano *et al*., 2020). Phytohormone metabolite contents confirmed an early and strong activation of plant phytohormone-based defense responses, while susceptibility of *P. radiata* was attributed to a delayed response to the fungus at the moment when symptoms became visible (Hernandez-Escribano *et al*., 2024). Zamora-Ballesteros *et al*. (2021) also found several up-regulated terpene synthases in *P. pinea* compared with *P. radiata* in their RNA-seq analysis.

This work focused on studying the resin-related response in *P. pinaster* and *P. radiata* under *F. circinatum* challenge to better understand the defense mechanisms against this pathogen. We compared the anatomical resin system and terpene profiles of these two hosts with different disease susceptibility, which we inoculated with two pathogen isolates of different virulence. We hypothesized that (i) resin duct size is negatively correlated with the greater resistance of *P. pinaster*; and (ii) differences in terpene composition contribute to the higher resistance of this species. We also used a previous transcriptomic study of the interaction between *P. pinaster* and *F. circinatum* (BioProject accession number PRJNA543723) to study the expression of terpene-related genes. This work provides background information on the underlying defense mechanisms against this disease and may assist in the development of PPC breeding programs.

## Materials and methods

### Plant material, inoculation, and treatments

Eleven-month-old *P. pinaster* (provenance Sierra Gredos ES-26-06) and *P. radiata* (provenance ES Monte Vasco Navarro) plants were purchased from a commercial nursery and maintained in a P2 biosafety greenhouse at 18–22 °C for the duration of the experiment.

Two *F. circinatum* isolates [isolate ID 7 (CECT20759 from the Colección Española de Cultivos Tipo, Valencia, Spain) and isolate ID 26 (Fungi collection at Instituto de Ciencias Forestales, INIA-CSIC, Madrid, Spain, available under request] were used for inoculation. These two isolates were chosen as representing the first and second most abundant haplotypes in Spain and have been previously characterized (Elvira-Recuenco *et al*., 2021) (Berbegal *et al*., 2013; Fariña-Flores *et al*., 2023). The isolates were collected in 2004–2005 from northern Spain, stored, grown for spore production, and spore suspensions were prepared as previously described (Elvira-Recuenco *et al*., 2021). The number of spores was quantified using a hemocytometer. Inoculations were performed on seedlings (Hernandez-Escribano *et al*., 2020) acclimated for 1 week before inoculation. Briefly, the first 2 cm of the shoot tip was cut, and a 2 μl drop of the spore suspension (1000 conidia) was deposited into the wound with a micropipette.

Seedlings of both pine species were subjected to the following treatments: inoculation with isolates 7 and 26 of *F. circinatum* (I7 and I26), mock inoculation with sterile water (MI), and unwounded plants (UW). The top 1.5 cm of the stem was cut and immediately frozen at −80 °C until use. For the anatomy study, five individual plants were subjected to each treatment. The top 2.5 cm was cut, and the needles were excised and frozen at −20 °C until use.

Final lesion length was measured before plants were collected. Seedlings were collected at 12 and 19 dpi, to ensure the maximum production of resin and TRDs.

### Anatomy of the resin system

Five seedlings per treatment and days post-infection of each species were sampled (a total of 80 seedlings). Transverse sections were taken from the area immediately below the lesion. Tissue pieces (2.5 cm long) were progressively dehydrated in successive ethanol series (70% to 100%) followed by xylene and embedded into paraffin blocks. Three 12 μm-thick transverse sections per sample were cut using a rotary microtome (RM2245, Leica, Heidelberg, Germany), stained with a mixture of safranin and astrablue (1:1), and mounted on permanent slides using Eukitt medium (Férriz *et al*., 2023). Slides were scanned at ×200 magnification with a digital camera (Leica DMC 5400) coupled to a microscope (Leica DM6 B) previously calibrated. We used ImageJ software (National Institutes of Health, Bethesda, MD, USA, https://imagej.net/ij) to measure different parameters in a randomly chosen quarter of each sample, taking into account the original scale of the images. The resin duct system in the xylem and cortex was characterized by (i) duct density, that is, the number of axial ducts (*N*) per area of either the xylem or cortex section (*N* mm^−2^); (ii) the mean diameter of ducts (including cell guards) (mm); and (iii) the total conductive area, calculated as *N* times the mean duct area in the total area of the transverse section (mm^2^).

### Gas chromatography–mass spectrometry analysis of the resin terpene content

For resin extraction, plant material was ground in liquid nitrogen using a mortar and pestle following Sampedro *et al*. (2010) with modifications. Two hundred milligrams of ground tissue was extracted for 24 h in 1 ml of hexane (Sigma-Aldrich, St Louis, MO, USA) at 4 °C using 0.025 mg ml^−1^ of heptadecanoic acid (Sigma-Aldrich), 0.0225 mg ml^−1^ of isobutylbenzene, and 0.025 mg ml^−1^ of heptadecane (Honeywell Fluka) as internal standards. An aliquot of this extract was used for analysis of volatile terpenes (MTs, STs, and DTs). For the analysis of DRAs, the remaining supernatant was dried under N_2_, and dissolved in methanol with tetramethylammonium hydroxide 1:10 (v/v) (Sigma-Aldrich) added as a methylation agent.

Terpenes were analysed, identified, and quantified by gas chromatography–mass spectrometry (GC-MS). An Agilent 6890N GC system (Agilent Technologies, Santa Clara, CA, USA) equipped with an Agilent 5973N quadrupole mass spectrometer was used for this analysis. A DB-5MS+DG capillary column (30 m×0.25 mm i.d., film thickness of 0.25 µm, Agilent) was used with helium as the carrier gas. Chromatographic conditions were those used by Fernández de Simón *et al*. (2017): injector temperature, 260 °C; column temperature, 60 °C during the split period (2 min, 5:1); heating, 4 °C min^−1^ to 272 °C (hold 10 min); constant flow rate, 1 ml min^−1^; MSD transfer line, 290 °C (MS source at 230 °C and MS quadrupole at 150 °C); and detection, which was performed in electron impact mode with ionization energy of 70 eV in the range of *m/z* 35–400. Compounds were identified by comparing their retention index and MS fragmentation patterns with an in-house reference library built with nearly 300 commercial standards and analysed under the same conditions: commercial MS libraries (Agilent Fiehn GC–MS Metabolomics RTL Library, Wiley7/Nist17 L GC/MS Library, and Adams, 1989), with matching of more than 95%, and literature data. For quantitative determinations, we used the internal standard method, with peak areas obtained from selected ion monitoring. Calibrations made with pure reference compounds analysed under the same conditions were used to calculate the concentrations of each analysed terpene.

### Statistical analysis

For analysis of the resin system, the effects of plant species (*P. radiata*, *P. pinaster*), treatments [inoculation with *F. circinatum* (I7 and I26), mock inoculation (MI), and unwounded (UW)], and dpi (12 and 19 dpi) were estimated by a mixed model with all variables considered fixed factors and including two- and three-term interactions, which were retained in the model when they were not significant. Pairwise comparisons of the least square means for all effects were performed using the Tukey test at a significance level of 0.05. For terpene profile analysis, three biological replicates composed each of a pool of eight plants were used and all statistical tests were performed on biological means. All analyses were performed with SAS Studio 3.8 (SAS Institute Inc., Cary, NC, USA). Similarly, the effects of pine species, the *F. circinatum* isolate, and dpi on the final lesion length were analysed. The overall metabolite concentrations within each group of MTs, DTs, STs, and DRAs were added and analysed similarly by a mixed model to determine the effects on terpene concentrations.

To identify terpenes that differed among the classes, univariate analysis of individual terpenes and multivariate analysis methods were applied. As a starting point, unsupervised principal component analysis (PCA) was used to reveal the class structure (groups defined by the combination of the variables in the experiment) (Worley and Powers, 2015). The supervised methods of partial least squares discriminant analysis (PLS-DA) and orthogonal PLS-DA (OPLS-DA) were used to identify the metabolite features highly correlated with class separation. The classes in the dataset were defined by species (*P. pinaster* and *P. radiata*), dpi (12 and 19), and treatment (UW, MI, I). PLS-DA model validation was assessed with the *Q^2^* statistic (Szymańska *et al*., 2012), which is an estimate of the predictive ability of the model and is calculated via cross-validation. When model quality was fulfilled, the variable importance in projection (VIP) score was chosen to select important variables in the PLS-DA model. When using an OPLS-DA model, discriminatory terpenes were selected on the S-plot. To avoid bias in the selection, only those terpenes that were statistically significant in class separation were retained (Trygg *et al*., 2007). In addition, we selected those with a fold change (FC) greater than 2. Analyses were performed with MetaboAnalyst 5.0 software (https://www.metaboanalyst.ca). The data were previously normalized to adjust for systematic differences via logarithmic transformation (base 10) and Pareto scaling. Hierarchical clustering heatmaps were generated using the Ward method with the Euclidean distance.

### Terpene-related genes from transcriptomic data of *P. pinaster*

To identify differentially expressed genes (DEGs) related to terpene biosynthesis, we examined the transcriptome of 7-month-old *P. pinaster* plants inoculated with *F. circinatum* (BioProject accession number PRJNA543723), which was analysed at 3, 5, and 10 dpi (Hernandez-Escribano *et al*., 2020). We performed BLAST similarity searches against the Kyoto Encyclopedia of Genes and Genomes (KEGG) database, and transcripts were positively identified when they met at least 70% similarity and 80% query coverage criteria.

## Results

### Lesion length

**Fig. 1.**
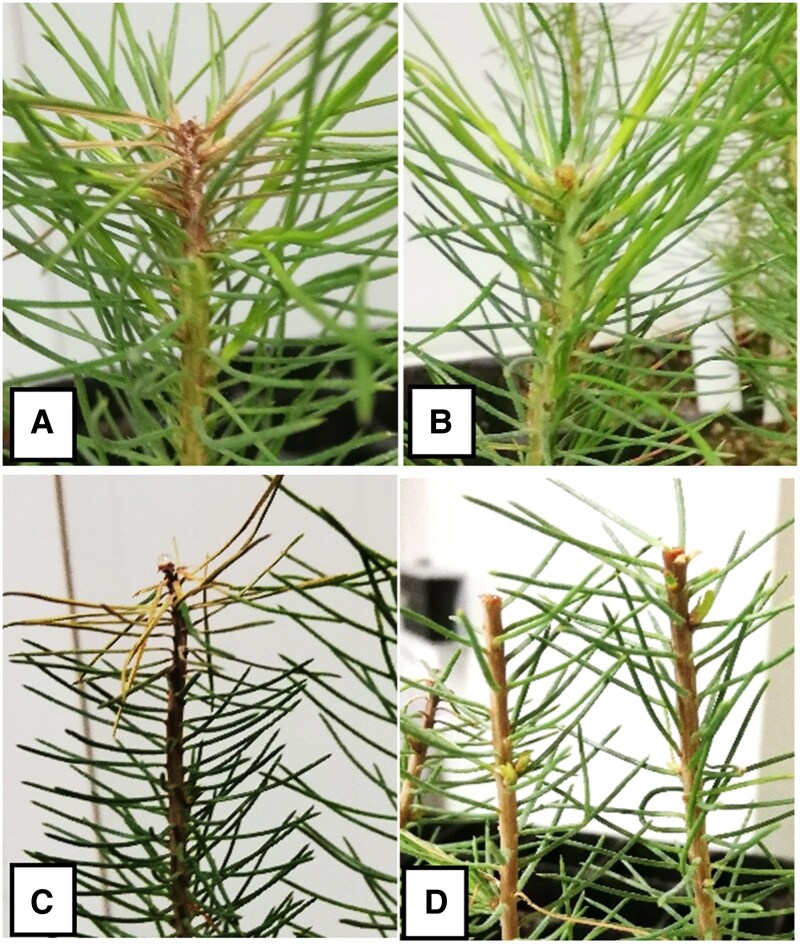
Symptoms at the shoot tip of *Pinus radiata* (A) and *P. pinaster* (C) inoculated with *Fusarium circinatum* and their respective mock-inoculated seedlings (B, D) at 19 d post-inoculation.

**Fig. 2.**
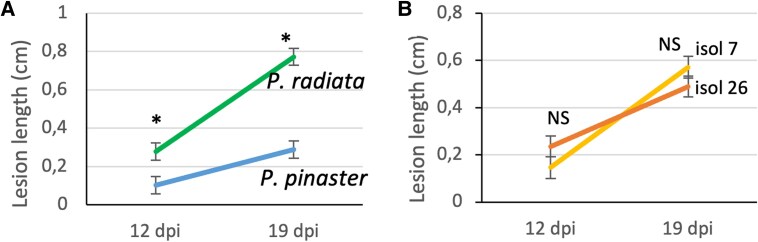
Lesion length on *Pinus pinaster* and *P. radiata* inoculated with two isolates of *Fusarium circinatum* (isolates 7 and 26), and measured at 12 and 19 days post-inoculation (dpi). Data are means of 24 replicates and error bars represent standard errors. For each dpi, differences in lesion length between species (A) (*P*<0.001) and between isolates (B) (not significant, NS) are shown. Asterisk indicates a significant difference (*P*<0.05) of the estimated least square means using a Tukey test.

Clear infection symptoms were visible at 19 dpi (Fig. 1). At 19 dpi, mean lesion length of *P. pinaster* and *P. radiata* was 0.29 cm and 0.77 cm, respectively (SE 0.044 cm), confirming that *P. pinaster* is more resistant than *P. radiata* (Fig. 2A). At 12 and 19 dpi lesions caused by I7 and I26 were similar in length (*P*=0.103 at 12 dpi and *P*=0.412 at 19 dpi), although at 19 dpi, lesions caused by I7 showed a trend toward being longer than those caused by I26 (Fig. 2B).

### Anatomy of the resin system

**Fig. 3.**
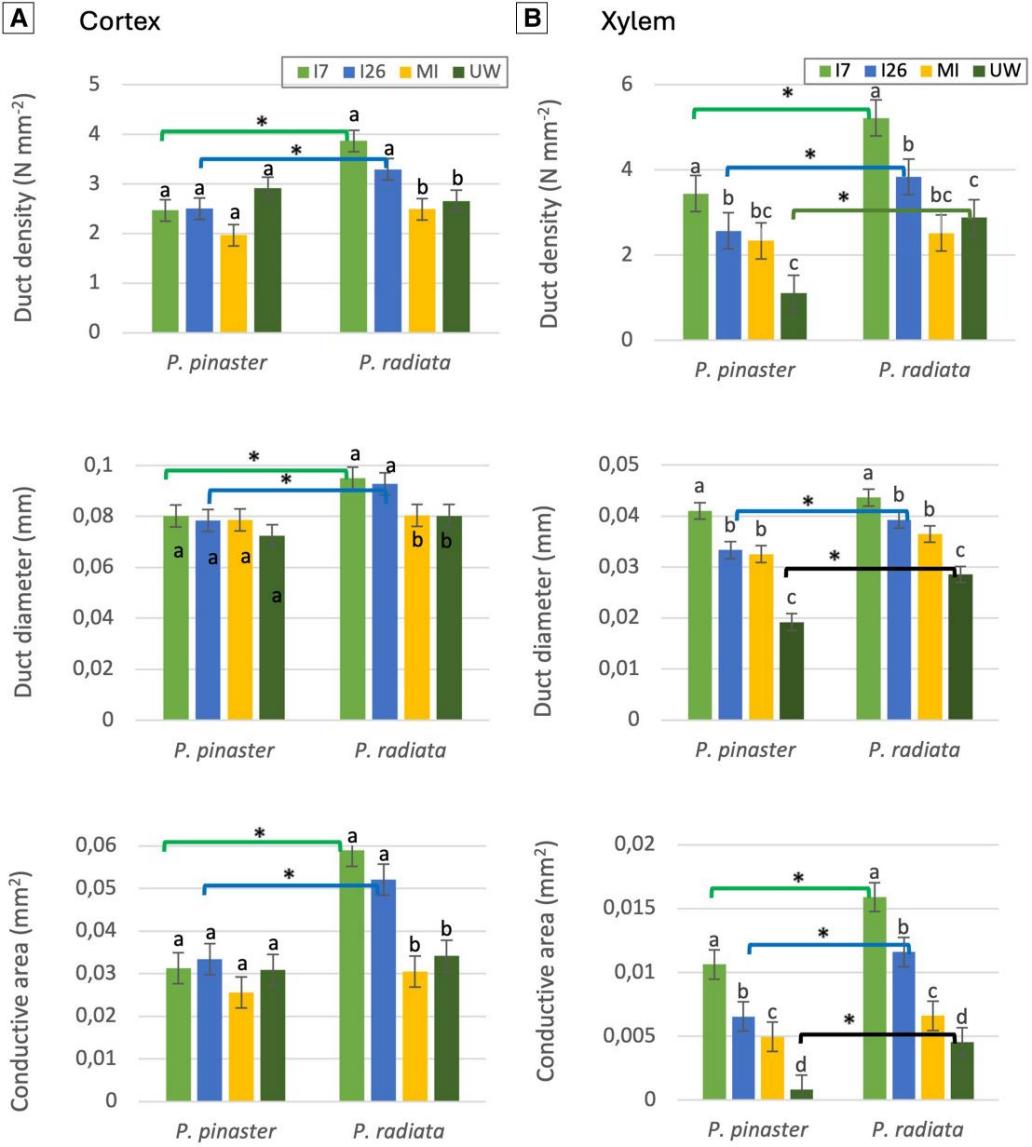
Resin duct features in the cortex (A) and xylem (B) of *Pinus pinaster* and *P. radiata* seedlings inoculated with two isolates of *Fusarium circinatum* [isolates 7 (I7) and 26 (I26)], measured at 12 and 19 d post-inoculation (bars represent means averaged across both time points for species and treatment groups). MI, mock inoculation; UW, unwounded. Data are mean of five replicates and the error bars represent standard errors. Estimated least square means were compared by Tukey test (*P*<0.05). For each species, means with the same letter are not significantly different. For each treatment, means linked by a line with an asterisk are significantly different between species.

In the UW treatment (Fig. 3), the constitutive resin ducts of *P. pinaster* and *P. radiata* differed in the xylem but not in the cortex. The mean values of all measured traits in the *P. radiata* xylem were significantly greater than those measured in the *P. pinaster* xylem; the *P. radiata* ducts in the xylem were wider (1.5-fold), their density was greater (2.6-fold), and the total conductive area was greater (5.6-fold).

MI treatment (wounding) did not increase resin duct formation in the cortex of *P. pinaster* or *P. radiata*, but it significantly increased the diameter and conductive area of the resin ducts in the xylem of both species compared with those of the unwounded plants (Fig. 3). However, these traits were not significantly different between species (Fig. 3).

Inoculation with *F. circinatum* isolates induced an increase in resin duct traits in the *P. radiata* cortex but not in that of *P. pinaster* (Fig. 3). Duct formation was due exclusively to the fungus (significant differences between I and MI, but similar between MI and UW), with increases in diameter, density, and conducive area of 17%, 39%, and 72%, respectively, compared with unwounded plants (Fig. 3). The most notable effect was the increment in the conductive area in the xylem of both species, where the two fungal isolates caused an increase compared with wounding (I7, 2.1-fold in *P. pinaster*, and 2.4-fold in *P. radiata*). The diameter and density of the induced resin ducts in the xylem were affected only by I7 compared with wounding (Fig. 3). The increase in diameter was 107% and 48% over wounded *P. radiata* and *P. pinaster* seedlings, respectively; density increased by 20% over wounded *P. radiata* and 26% over wounded *P. pinaster* seedlings.

**Fig. 4.**
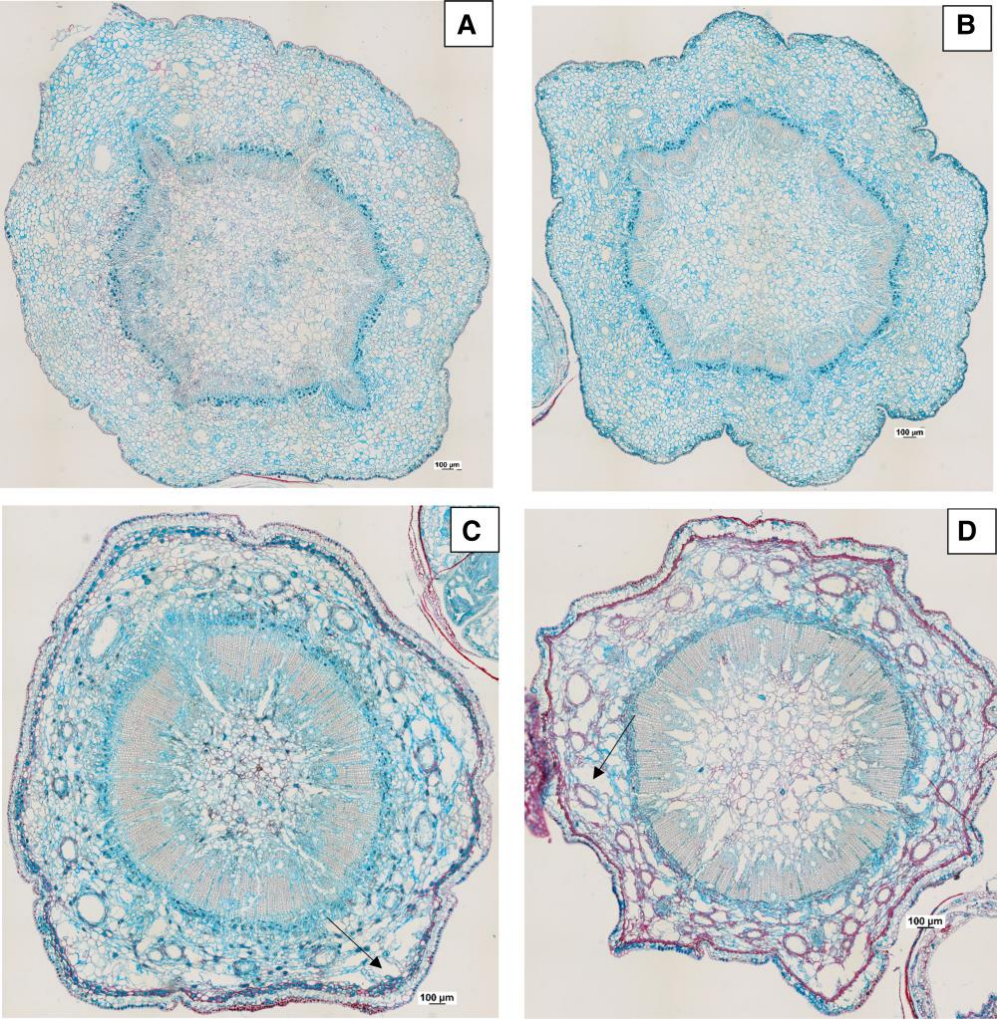
Examples of transverse sections of the shoot top (the first 2 cm of the shoot tip was previously removed for inoculation) showing resin ducts in the cortex and the xylem of *Pinus pinaster* (A, B) and *P. radiata* (C, D) seedlings inoculated with two isolates [isolates 7 (B, D) and 26 (A, C)] of *Fusarium circinatum* at 19 d post-inoculation. Note the disruption in the cortical parenchyma (arrows) of *P. radiata*.

Microscopic examination of transverse sections revealed that infection in pathogen-inoculated *P. radiata* seedlings caused tissue disruption in the cortex. In this zone, the parenchyma of the seedlings degraded, resulting in large gaps. In contrast, *P. pinaster*-infected seedlings preserved their structure (Fig. 4).

### Terpene response

A total of 182 terpenes were detected in the resin extracted from the stem tips (the dataset is available at Zenodo, https://doi.org/10.5281/zenodo.15582968; Fariña-Flores *et al*., 2025), and only four of them were unidentified. Eight metabolites were discarded because an analysis of variance (ANOVA) on individual compounds revealed no differences among factors. Therefore, the terpene profiles were composed of 174 metabolites in total: 54 MTs, 39 STs, 34 DTs, and 47 DRAs.

### Constitutive resin composition

**Fig. 5.**
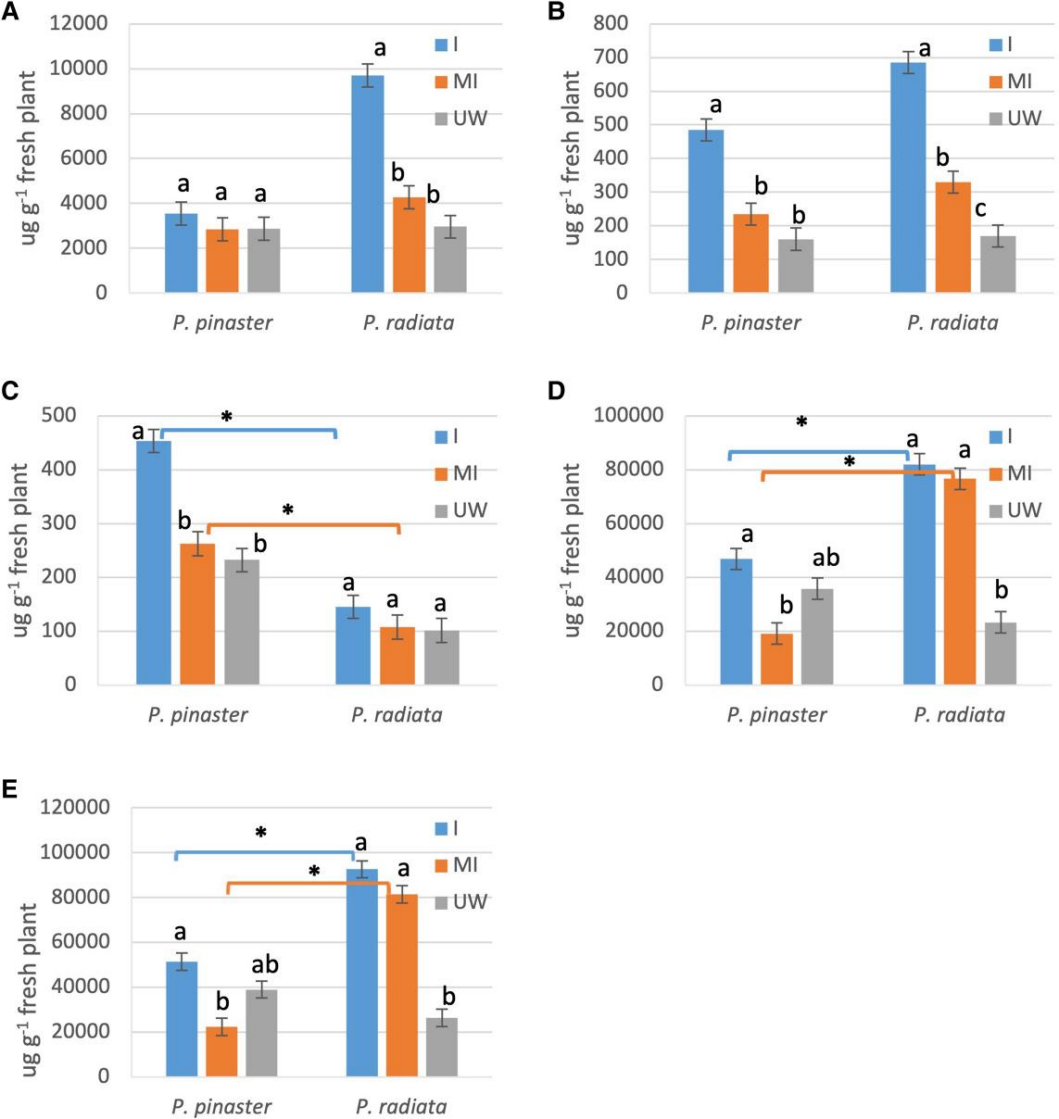
Total content (µg g^−1^ fresh plant) of the terpene groups in *Fusarium circinatum*-inoculated (I), mock-inoculated (MI), and unwounded (UW) *Pinus pinaster* and *P. radiata* seedlings. (A) Monoterpenes; (B) diterpenes; (C) sesquiterpenes; (D) diterpene resin acids; and (E) total. Data are the mean and SE of three replicates, pooled over isolate in the I treatment. For each terpene group and species, means with the same letter are not significantly different by Tukey test. Bars linked by a line with an asterisk are significant treatments between species.

The total amount of constitutive resin in *P. pinaster* was 39 017±4739.9 µg g^−1^ plant, and was significantly higher (*P*=0.03) than that in *P. radiata*, which was 26 453±2347.7 µg g^−1^ plant (UW treatment, Fig. 5). The resin of both species was composed mainly of DRAs, which represented approximately 90% of the total terpene content. Among the neutral terpenes, the MT group had the greatest abundance (Fig. 5). Individual terpene contents were studied using an OPLS-DA model, from which terpenes differing between *P. pinaster* and *P. radiata* were selected in an S-plot (Supplementary Fig. S1; Supplementary Table S1). Resin of *P. radiata* contained elevated MTs (ranging from 13- to 774-fold higher), particularly phenylethyl butyrate, citronellal, and citronellol. Resin of *P. pinaster* showed enriched STs, especially valencene (1071-fold higher in *P. pinaster*).) One ST [abieta-8(14), 13(15)-diene] appeared exclusively in induced resin of both species.

### Changes in the terpene content in response to wounding and infection with *Fusarium circinatum*

#### Pine species responded differently in terms of terpene content

For each species, pathogen infection and wounding had different effects on the content of terpenes (MTs, DTs, STs, and DRAs) (treatment×species interaction terms significant for all terpene groups except DTs). No differences in terpene content were found between the fungal isolates. *Pinus pinaster* seedlings increased DT (2-fold) and ST (1.7-fold) content only with *F. circinatum* infection compared with wounded plants (Fig. 5), while DRA content responded to wounding alone (2.4-fold relative to unwounding) (Fig. 5). *Pinus radiata*-infected plants increased MT (2.3-fold) and DT (2-fold) contents; the DRA content group increased (1.4-fold) with wounding (Fig. 5).

Total resin was abundantly induced in *P. radiata*, after either wounding (three times more) or infection (3.6 times more than unwounding) (Fig. 5E). Comparing infected species, *P. radiata* increased MTs (2.7-fold), DTs (1.4-fold), and DRAs (1.7. fold), while *P. pinaster* increased ST content by 3.1-fold (Fig. 5). The content of neutral terpenes (not including DRAs) was 2.4 times greater in *P. radiata*. Temporal analysis revealed continued DT accumulation (from 12 to 19 dpi) (dpi×treatment interaction term was significant) in both infected species, while STs increased over time exclusively in *P. pinaster*. Wounded seedlings did not vary in content in any group from 12 to 19 dpi.

#### Terpene profiles are grouped by species, type of challenge affecting seedlings, and time

**Fig. 6.**
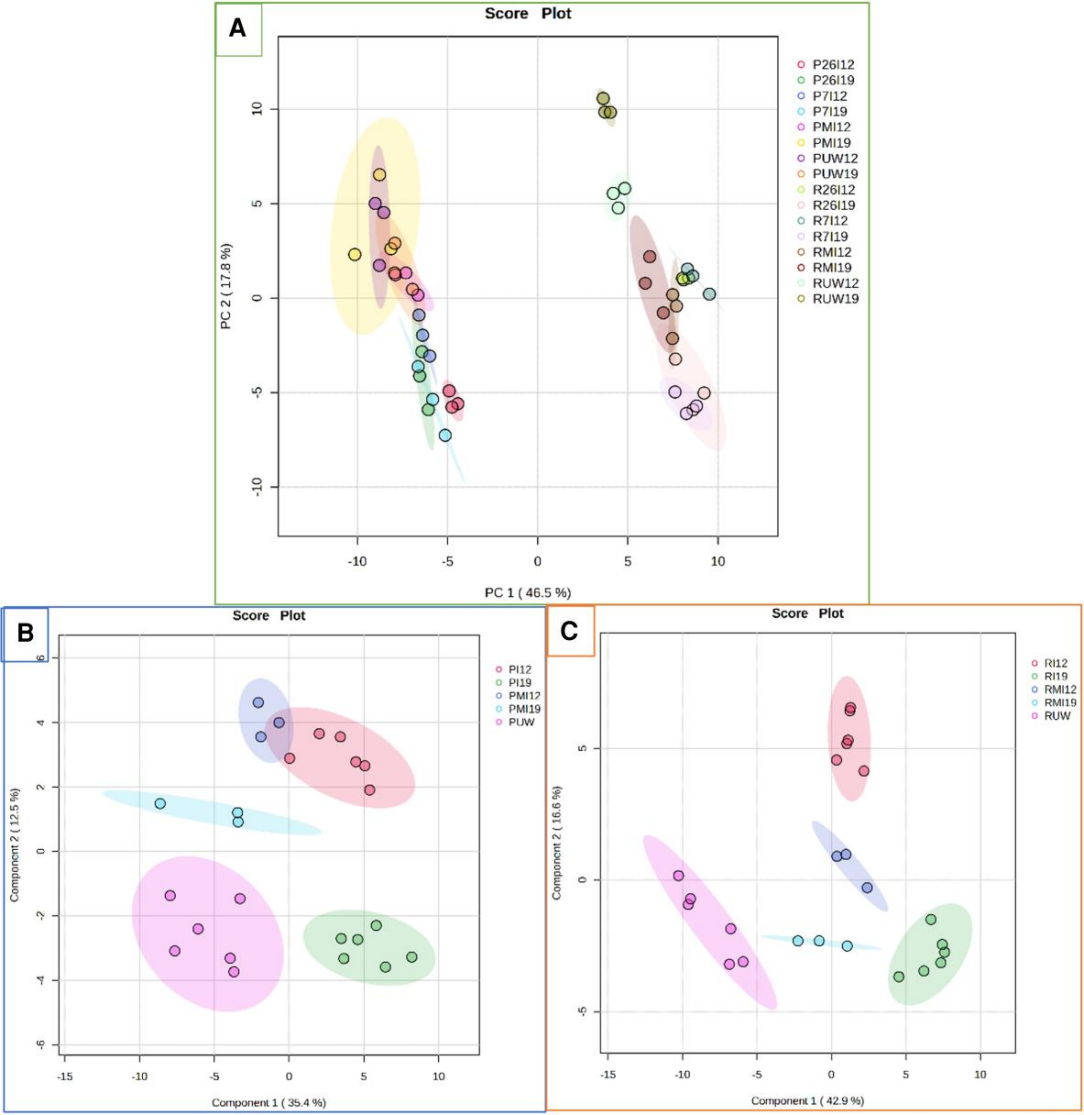
Principal component analysis (PCA) (A) and partial least squares discriminant analysis (PLS-DA) (B, C) score plots of normalized data from terpene profiles by GC-MS on seedlings of *Pinus pinaster* (B) and *P. radiata* (C). PCA was performed on the overall data, and PLS-DA was performed separately on the *P. pinaster* (P) and *P. radiata* (R) data. 7I and 26I indicate data from seedlings inoculated with *Fusarium circinatum* isolates 7 and 26, collected at 12 (7I12, 26I12) and 19 (7I19, 26I19) days post-inoculation (dpi); MI12 and MI19 are from mock-inoculated seedlings at 12 and 19 dpi; UW12 and UW19 are from unwounded plants at 12 and 19 dpi, UW from pooled data.

Terpene profiles were explored using PCA. The first two components (PC1 and PC2) explained 65% of the variance, and the PCA score plot (Fig. 6A) indicated a major effect of species associated with PC1 (47% variance). The close grouping of the biological replicates by treatment indicated good reproducibility. Metabolite features highly correlated with class separation were only achieved when a PLS-DA model was adjusted separately by species, verifying a specific terpene profile for each species (Fig. 6B, C). Moreover, to make the adjustment it was necessary to use pooled data from unwounded seedlings at 12 and 19 dpi, and combined data from both *F. circinatum* isolates, resulting in a total of five classes (named I12 and I19, MI12 and MI19, and UW). Only then were the validation parameters for the PLS-DA models acceptable (for *P. pinaster*, *R*^2^=0.91, *Q*^2^= 0.76 with four components, *P*=0.001; and for *P. radiata*, *R*^2^=0.84, *Q*^2^=0.74 with two components, *P*<0.0001).

**Fig. 7.**
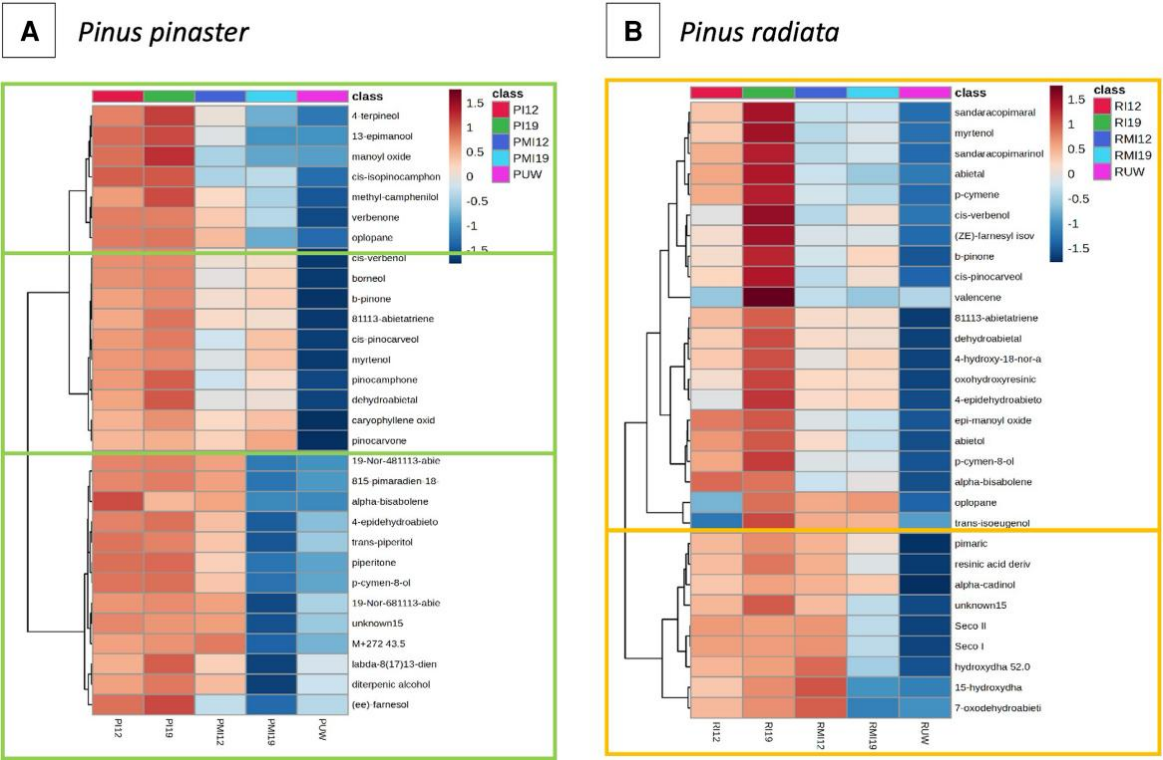
Hierarchical clustering heatmaps with the 30 top terpenes based on variable importance in projection (VIP) from partial least squares discriminant analysis (PLS-DA) models for *Pinus pinaster* (A) and *P. radiata* (B). PLS-DA validation parameters are *R*^2^=0.9, *Q*^2^=0.75, *P*=0.001 for *P. pinaster* and *R*^2^=0.85, *Q*^2^=0.74, *P*<0.0001 for *P. radiata*. I12 and I19 data are from seedlings inoculated with *Fusarium circinatum* and collected at 12 and 19 days post-inoculation (dpi); MI12 and MI19 data are from mock-inoculated seedlings; UW pooled data are from unwounded plants at 12 and 19 dpi. Numbers in the figure indicate clusters obtained with the Ward method and Euclidean distance. The numerical terpene contents are shown in Supplementary Table S2.

From the respective PLS-DA model, the most important metabolite features discriminating the five classes were chosen using the VIP score. The top 30 metabolites (with VIP values >1.30) (Supplementary Table S2) were used to construct heatmaps of the normalized terpene content (Fig. 7). An increase in terpene content in response to wounding or infection occurred earlier in *P. pinaster* than in *P. radiata* (indicated by greater red color brightness, Fig. 7), and this level was maintained over time in infected plants. The *P. pinaster* heatmap (Fig. 7A) grouped terpenes into three main clusters, showing major differences due to wounding (mock inoculation). Of the 30 terpenes included in the heatmap, 15 were MTs (although present at low concentrations), and none were DRAs (Supplementary Table S2). Several terpenes (*p*-cymen-8-ol, α-bisabolene, 8,15-pimaradien-18-al, β-pinone, *cis*-verbenol, oplopane, verbenone, and *cis*-isopinocamphone) were present at low levels (nearly zero) in the UW seedlings (Supplementary Table S2). The highest DT content in the inoculated class was 19-Nor-4,8,11,13-abietatetraene, followed by borneol (MT) (Supplementary Table S2). The *P. radiata* heatmap (Fig. 7B) revealed two clusters that differed in response to wounding, with terpenes being more abundant in cluster 2 at 12 dpi. Unlike *P. pinaster*, some terpenes in this heatmap were in the DRA group, and they reached the highest content in the inoculated classes (in the range of 47.3–2270.4 µg g^−1^ plant) (Supplementary Table S2). For neither of these two species did the clusters include terpenes from a single group.

#### Discriminatory terpenes in infected seedlings and over time

Four OPLS-DA models were built (one per species and dpi) and the most relevant terpenes that characterized the infected plants with respect to the wounded plants were selected using S-plots. Additionally, two temporal models (one per species) identified differential terpenes over time.

In *P. pinaster*, we identified nine terpenes at 12 dpi and eight at 19 dpi that were significantly more abundant in the presence of *F. circinatum* (with fold-changes ranging from 6 to 12 at 12 dpi and 2 to 5 at 19 dpi) (Supplementary Table S3). Other terpenes [thymol and citronellol (MTs); farnesyl isovalerate and α-cadinene (STs); and 19-Nor-6,8,11,13-abietatetraene and labda-8(17),13-dien-15-al (DTs)] displayed high OPLS weights at 19 dpi, but lacked statistical significance between inoculated and mock-inoculated plants. *Pinus radiata* exhibited contrasting patterns. At 12 dpi, three terpenes [13-epimanool, resinic 49.4, and hydroxyabietic 51.8 (the last two not fully identified)] increased under infection (FC>10), while all other discriminatory metabolites (primarily STs and DTs) significantly decreased (FC<1) (Supplementary Table S3). At 19 dpi, all identified terpenes accumulated in infected seedlings, with the DRA 7-oxodehydroabietic showing exceptional induction (FC=125). Notably, several terpenes displayed opposite responses between species. Abieta-8(14),13(15)-diene, pimarol, 8,15-pimaradien-18-al, and labda-8(17),13*E*-dien-15-al increased in infected *P. pinaster* seedlings (FC>1) but decreased in *P. radiata* (FC<1) (Table 1). Temporal differences also emerged: 15-hydroxy and 7-oxo dehydroabietic acids (DRAs) and *cis*-isopinocamphone (MT) appeared at 12 dpi in infected *P. pinaster*, but not until 19 dpi in *P. radiata* plants (Table 1). An ANOVA for comparing treatment means (I, MI, and UW) of discriminatory terpenes revealed that some of them also changed in response to wounding (Table 2) and showed that not only infection but also wounding of *P. radiata* at 12 dpi inhibited several of those terpenes.

**Table 1.** Fold change of terpenes discriminating *F. circinatum*-infected from wounded seedlings for *Pinus pinaster* and *P. radiata* at 12 and 19 dpi

Terpene	Group	FC (I/MI^*a*^)	Log_2_ FC
*P. pinaster*, 12 dpi			
*cis*-Isopinocamphone	MT	10.7	3.417
15-Hydroxydehydroabietic	DRA	8.7	3.120
Abieta-8(14),13(15)-diene	DT	7.7	2.945
7-Oxodehydroabietic	DRA	6.6	2.715
*P. pinaster*, 19 dpi			
Pimarol	DT	4.5	2.185
Labda-8(17),13*E*-dien-15-al	DT	3.1	1.631
8,15-Pimaradien-18-al	DT	2.3	1.193
*P. radiata*, 12 dpi			
8,15-Pimaradien-18-al	DT	0.16	−2.606
Labda-8(17),13*E*-dien-15-al	DT	0.14	−2.851
Abieta-8(14),13(15)-diene	DT	0.12	−3.031
Pimarol	DT	0.11	−3.164
*P. radiata*, 19 dpi			
7-Oxodehydroabietic	DRA	125.1	6.967
15-Hydroxydehydroabietic	DRA	17.8	4.155
*cis*-Isopinocamphone	MT	8.0	3.008

Terpenes were selected from their respective orthogonal partial least squares discriminant analysis models, and only terpenes common to both species are listed. The complete list is in Supplementary Table S3. dpi, days post-inoculation; DT, neutral diterpene; DRA, diterpene resin acid; FC, fold change; MT, monoterpene.

^*a*^I, infected; MI, wounding.

**Table 2.** Normalized means of terpene contents (µg g^−1^ fresh plant) of inoculated (I), mock-inoculated (MI), and unwounded (UW) seedlings that discriminate between groups of *Fusarium circinatum*-infected and mock-inoculated seedlings and are common to both pine species

Terpene	I	MI	UW
*Pinus pinaster*, 12 dpi			
*cis*-Isopinocamphone	−0.0102 a	−1.0089 b	−1.5118 b
15-Hydroxydehydroabietic	0.6247 a	−0.2693 b	−1.8392 c
Abieta-8(14),13(15)-diene	1.2092 a	−0.2808 b	−0.2808 b
7-Oxodehydroabietic	0.3319 a	−0.4084 b	−1.8599 c
*Pinus pinaster*, 19 dpi			
Pimarol	0.3922 a	−1.1364 b	0.0016 a
Labda-8(17),13*E*-dien-15-al	1.1284 a	−1.4121 b	0.6503 a
8,15-Pimaradien-18-al	0.6186 a	−1.3318 b	−1.3318 b
*Pinus radiata*, 12 dpi			
8,15-Pimaradien-18-al	−1.3318 c	0.7695 b	0.4655 a
Labda-8(17),13*E*-dien-15-al	−1.4121 c	−0.1342 b	−0.4386 a
Abieta-8(14),13(15)-diene	−0.2808 b	1.2318 a	−0.2808 b
Pimarol	−1.1364 c	0.0120 b	0.6459 a
*Pinus radiata*, 19 dpi			
7-Oxodehydroabietic	1.2022 a	−0.9104 b	−0.6347 b
15-Hydroxydehydroabietic	0.8429 a	−0.5088 b	−0.3618 b
*cis*-Isopinocamphone	1.3846 a	0.5109 b	0.2646 b

For each terpene, means with the same letter are not significantly different by Tukey test.

Temporal analysis identified distinct patterns in infected *P. pinaster*: citronellic acid, 7-oxodehydroabietic acid, and abieta-8(14),13(15)-diene were abundantly produced at 12 dpi (FC>1) (Supplementary Table S4), while other terpenes (including some DRAs not fully identified), accumulated more at 19 dpi (FC<1). In contrast, all discriminatory terpenes identified in *P. radiata* increased from 12 to 19 dpi (FC<1) (Supplementary Table S4).

### Identification of terpene-related genes from transcriptomic data of *Pinus pinaster* and *fusarium circinatum*

A total of 59 DEGs related to terpene biosynthesis were identified from the transcriptome dataset (Hernandez-Escribano *et al*., 2020) (BioProject accession number PRJNA543723) of *P. pinaster* under *F. circinatum* infection at 3, 5, and 10 dpi, determined previously using a dual RNA-seq assay (Supplementary Table S5).

**Fig. 8.**
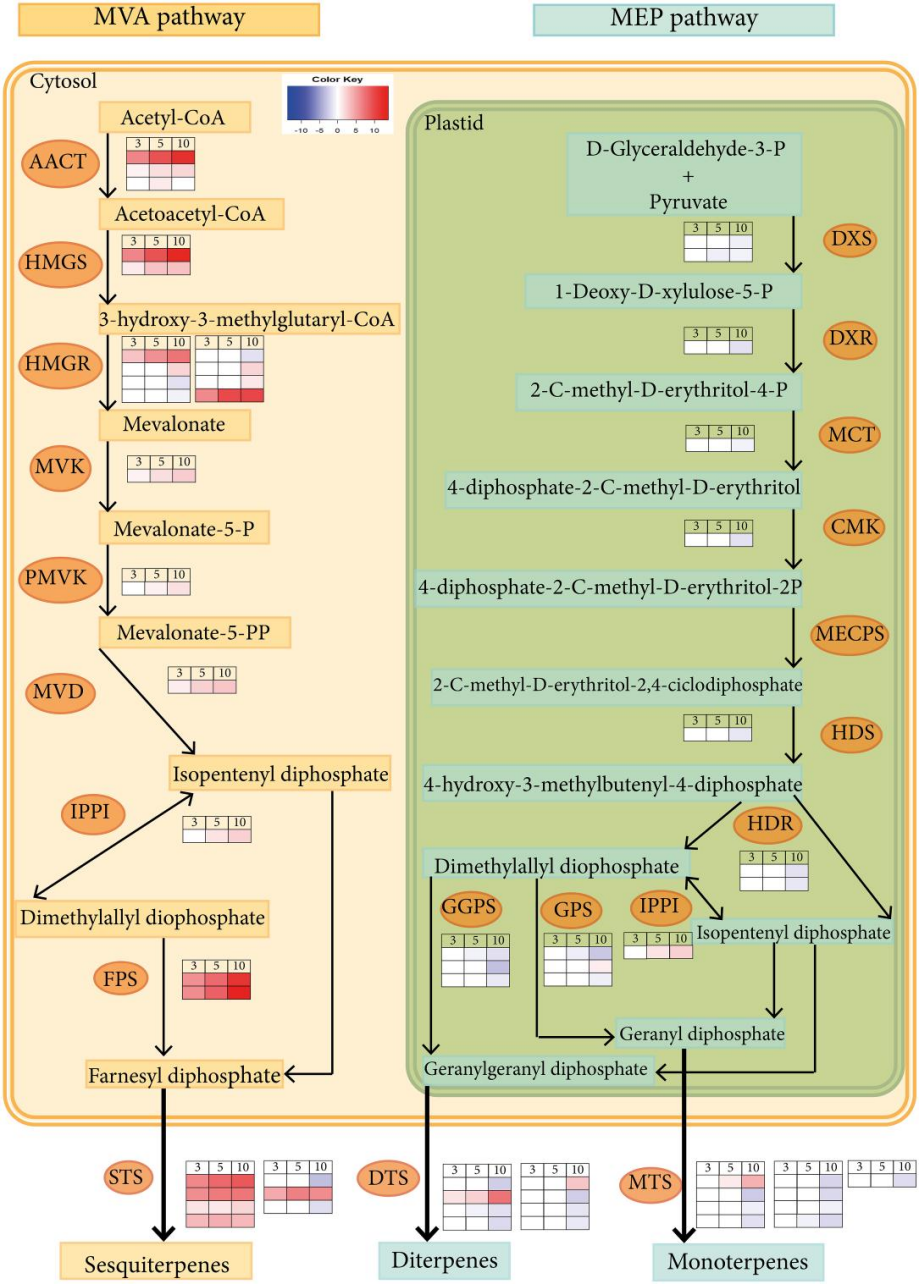
Differentially expressed genes of the corresponding enzymes in the MVA and MEP pathways identified from the transcriptome of *Pinus pinaster* under *Fusarium circinatum* infection. The heatmaps show the relative gene expression levels at 3, 5, and 10 d post-inoculation. AACT, acetyl-CoA C-acetyltransferase; CMK, 4-(cytidine 5′-diphospho)-2-C-methyl-D-erythritol kinase; DTS, diterpene synthases; DXR, 1-deoxy-D-xylulose 5-phosphate reductoisomerase; DXS, 1-deoxy-D-xylulose-5-phosphate synthase; GGPS, geranylgeranyl-diphosphate synthase; GPS, geranyl-diphosphate synthase; HDR, 4-hydroxy-3-methylbut-2-en-1-yl diphosphate reductase; HDS, (*E*)-4-hydroxy-3-methylbut-2-enyl-diphosphate synthase (ferredoxin); HMGR, hydroxymethylglutaryl-CoA reductase (NADPH); HMGS, hydroxymethylglutaryl-CoA synthase; FPS, farnesyl diphosphate synthase; IPPI, isopentenyl-diphosphate delta-isomerase; MCT, 2-C-methyl-D-erythritol 4-phosphate cytidylyltransferase; MECPS, 2-C-methyl-D-erythritol 2,4-cyclodiphosphate synthase; MEP, methyl-erythritol phosphate; MTS, monoterpene synthases; MVA, mevalonate; MVD, diphosphomevalonate decarboxylase; MVK, mevalonate kinase; PMVK, phosphomevalonate kinase; STS, sesquiterpene synthases.

The results suggested that *F. circinatum* induced the expression of genes involved in the MVA pathway of terpenoid backbone biosynthesis and sesquiterpene-related genes and the repression of genes associated with the MEP pathway, as well as MT- and DT-related genes (Fig. 8; Supplementary Table S5). Nineteen genes related to the MVA pathway were differentially expressed, of which 16 were up-regulated and three were down-regulated at 10 dpi. Thirteen genes in the MEP pathway were down-regulated at 10 dpi (Fig. 8; Supplementary Table S5), and only one gene was up-regulated. Eight genes involved in MT biosynthesis and seven involved in DT biosynthesis were down-regulated at 10 dpi, while five of the eight ST-related genes were up-regulated at all dpi.

With respect to the pathogen, one terpene synthase gene was differentially expressed in the *F. circinatum* dataset at 10 dpi. This gene was annotated as pentanelene synthase, which catalyses the conversion of 2-*trans*,6-*trans*-farnesyl diphosphate to the sesquiterpene pentalene.

## Discussion

### Anatomical structure and resin content

Resin duct formation and resin production generally confer protection to pines against biotic and abiotic stresses (Krokene *et al*., 2003; Kim *et al*., 2010; Krokene and Nagy, 2012; Celedon and Bohlmann, 2019; Chen *et al*., 2019). However, our results reveal contradictory findings: the *F. circinatum*-susceptible species *P. radiata* (i) induces larger resin ducts, and (ii) produces more resin than the moderately resistant *P. pinaster*.

#### Resin ducts induced by *F. circinatum* are larger in the susceptible species *P. radiata*

Although larger resin ducts are traditionally associated with greater resistance (Nagy *et al*., 2006; Ferrenberg *et al*., 2014; Vázquez-González *et al*., 2020), our data demonstrate the opposite. This result aligns with previous studies on pathogens that colonize through resin ducts, such as the pinewood nematode *Bursaphelenchus xylophilus* (Kawaguchi, 2006; Nunes da Silva *et al*., 2015; Rodríguez-García *et al*., 2023). *Fusarium circinatum* specifically attacks resin ducts, growing in both constitutive and induced systems (Martín-Rodrigues *et al*., 2013). During colonization, resin-producing epithelial cells become hypertrophied and tylosoids are formed. Fungal hyphae are located around and inside the xylem resin ducts, invading through the intercellular spaces of the cortex and advancing until they reach the pith (Barrows-Broaddus and Dwinell, 1983; Martín-Rodrigues *et al*., 2013). The pathogen colonizes the host vertically through the cortex and phloem (consistent with a visible external lesion), through the xylem using the resin ducts and tracheids, and through the pith (Martín-Rodrigues *et al*., 2013). This colonization strategy explains why a more developed duct system favors disease progression rather than preventing it.

We found that *F. circinatum* inoculation induced resin duct formation in both the xylem and the cortex of susceptible seedlings (Fig. 3), contrary to previous studies reporting induction only in xylem, in response to stresses (Franceschi *et al*., 2005; Celedon and Bohlmann, 2019) or after application of methyl jasmonate to pines (López-Villamor *et al*., 2021). However, that pattern was previously observed in susceptible species such as *Pinus virginiana* Mill. and *Pinus elliottii* Engelm., whose induced ducts in the cortex were wider than those of *Pinus taeda* L. and *Pinus serotina* Michx. (Barrows-Broaddus and Dwinell, 1983). Our results confirm and extend these observations: induced ducts in the cortex of susceptible species are not only wider but also show greater density and total conductive area.

The more virulent isolate (I7) induced larger resin ducts in the xylem of both species, confirming the close relationship between the duct system and host colonization. This is the first anatomical study comparing isolates of *F. circinatum* with different virulence. Inoculation with the more virulent isolate caused a larger lesion than that caused by I26 at 19 dpi, although this difference was not significant. The virulence of I7 and I26 has been compared previously (Iturritxa *et al*., 2012, 2013; Elvira-Recuenco *et al*., 2021), showing that the lesion length measured over a longer period, spore germination, and spore production were greater for the more virulent isolate. Transcriptome analysis during the interaction of *F. circinatum* with *P. pinaster* (Hernandez-Escribano *et al*., 2020) revealed the activation of genes associated with complex phytohormone signaling involving jasmonic acid, ethylene, and salicylic acid. Moreover, it was suggested that *F. circinatum* manipulates the host phytohormone balance through the expression of fungal genes related to those phytohormones (Hernandez-Escribano *et al*., 2020). The formation of TRDs is an induced response involving changes in cell division and differentiation (Franceschi *et al*., 2005; Krokene and Nagy, 2012), in which jasmonate and ethylene signaling are implicated (Wang *et al*., 2002; Wasternack, 2007; Schmidt *et al*., 2011). Our results in *P. pinaster* are compatible with the hypothesis that hormones are manipulated by the pathogen, suggesting that different pathogen genotypes could differentially modulate duct formation.

#### Resin quantity induced by *F. circinatum* is greater in the susceptible species *P. radiata*

The increase in duct formation in *P. radiata* was accompanied by greater induced resin production (Fig. 5). Previous studies showed that external resin flow following inoculation with *F. circinatum* was greater in susceptible species (Enebak and Stanosz, 2003; Kim *et al*., 2010), suggesting a lack of protection conferred by the induced resin against this pathogen. We quantified the total increase in resin but separately from the wounding response, and confirmed that terpene abundance is not related to increased resistance to *F. circinatum*.

*Fusarium circinatum* shows enhanced growth in susceptible plants associated with increased resin duct formation and increased resin production. Some invading organisms, especially bark beetles, can overcome resin-based defenses transforming terpenes into pheromones (Phillips and Croteau, 1999; Keeling and Bohlmann, 2006). Other organisms, such as beetle-associated fungi growing in environments rich in terpenes, use terpenes to promote their growth (Kusumoto *et al*., 2014; Mason *et al*., 2015). *Fusarium circinatum in vitro* growth increased with resin from *P. radiata* and *P. pinaster* (Blomquist *et al*., 2010; Slinski *et al*., 2015), and its growth was 40% more in the presence of induced *P. radiata* resin than in the constitutive (Blomquist *et al*., 2010). Considering that induced resin ducts and resin production provide no further protection against infection, and that *F. circinatum* growth is enhanced by resin, it can be concluded that *F. circinatum* is a specialist pathogen that exploits resin-based defense mechanisms to its benefit.

### Terpene profiles and gene expression

The resin contents induced by infection with *F. circinatum* differed between the two species. The higher content of STs at 12 dpi achieved by *P. pinaster* is supported by the DEGs identified from the transcriptomic data. Consistently, genes for all enzymes involved in the MAV pathway (biosynthesis of ST precursors) (Fig. 8) were overexpressed (Supplementary Table S5), and those involved in the MEP pathway (biosynthesis of MT and DT precursors) were down-regulated. In addition to the high ST content, *F. circinatum* induced increases in DT, and in DTs and MTs in *P. radiata*. These results contradict the previous work of Lombardero *et al*. (2019), who found that *F. circinatum* induced changes in DT and MT content in *P. radiata* but not in *P. pinaster* when studying plant defenses mediated by the interaction between *F. circinatum* and *T. piniperda*. Methodological differences (such as inoculation, chemical, and analytical) could explain the variations in the presented results. Constitutive resins of both species were similar in total content but differed in terpene distribution, resulting in species-specific profiles. In response to wounding and infection, both species underwent quantitative and qualitative changes that affected the relative composition of individual terpenes. We identified a set of terpenes common to *P. radiata* and *P. pinaster* highly correlated with the differentiation of infected plants from mock-inoculated plants (Table 1). Four DTs [abieta-8(14),13(15)-diene, pimarol, 8,15-pimaradien-18-al, and labda-8(17),13*E*-dien-15-al] were more abundant in infected *P. pinaster*, which could partially explain its greater resistance.

The greater resistance of *P. pinaster* is also explained by a more rapid response to *F. circinatum* infection. This is illustrated in the heatmap (Fig. 7), which shows that terpene increase occurs earlier in *P. pinaster* than in *P. radiata* and is maintained over time. An earlier response is also shown when comparing content of common terpenes (Table 1): in infected *P. pinaster*, terpene content increased at 12 dpi, while in infected *P. radiata*, this increase did not occur until 19 dpi. An earlier response is especially evident when comparing terpenes discriminating between 12 and 19 dpi (Supplementary Table S4): in *P. radiata*, all identified terpenes were more abundant at 19 dpi (FC<1), indicating a slow response, while in *P. pinaster* three terpenes were already more abundant at 12 dpi (citronellic acid, 7-oxodehydroabietic acid, and abieta-8(14),13(15)-diene; the last two terpenes were also significant in infected *P. pinaster*, confirming their involvement in disease resistance). Transcriptomic studies have consistently linked early phytohormone response with greater resistance in pine species such as *P. tecunumanii* (Visser *et al*., 2019), *P. pinea* (Zamora-Ballesteros *et al*., 2021), and *P. pinaster* (Hernandez-Escribano *et al*., 2020). Auxin-, ethylene-, jasmonic-, and salicylic-mediated defenses are likely needed for resistance (Visser *et al*., 2019; Hernandez-Escribano *et al*., 2020). Our results suggest that early biosynthesis of specific terpenes equally contributes to host resistance.

We detected the DT abieta-8(14),13(15)-diene only in induced resin from infected *P. pinaster* and wounded *P. radiata*, but not in constitutive resin. No studies are known that identify terpenes in the induced resin previously absent in the constitutive resin, but most work addressing changes in terpene content in response to stresses focuses on the variation of specific terpenes (Lombardero *et al*., 2019; Kopaczyk *et al*., 2020; Trujillo-Moya *et al*., 2022) rather than studying terpene profiles. DTs play an important role in plant defense, since they are toxic to microorganisms and arthropods (López-Goldar *et al*., 2020) and participate in the biosynthesis of DRAs. The mode of action of some DTs has been described. For example, the labdane-type (11*E*,13*E*)-labda-11,13-diene-8α,15-diol contributes to the activation of pathogen- or wound-induced reactions in tobacco leaves (Gnanasekaran *et al*., 2015).

In summary, this study demonstrated that the defensive response to *F. circinatum* in pine seedlings increased induced resin duct formation in both the cortex and the xylem of *P. radiata*, being larger in diameter, density, and total conductive area than in *P. pinaster.* Although resin duct traits are expected to be positively related to resistance, we confirm they are larger in susceptible species, a result consistent with pathogens such as *F. circinatum* that use resin ducts to colonize the host.

Comparison of terpene profiles between *P. pinaster* and *P. radiata* revealed differences in constitutive composition, and in response to wounding and infection with *F. circinatum*. We identified four DTs produced more abundantly in infected *P. pinaster* that could be involved in plant defense. Differences between species suggest a more rapid increase of some terpenes in *P. pinaster*, which could reflect a higher disease resistance. The transcriptome of the *P. pinaster*-*F. circinatum* interaction showed up-regulation of MVA pathway genes and sesquiterpene synthases, supporting the increased ST content identified in infected plants. This study provided abundant information on terpene contents that contributes to understanding disease response.

## Supplementary Material

erag023_Supplementary_Data

## Data Availability

The terpene profile is available at Zenodo https://doi.org/10.5281/zenodo.15582968 (Fariña-Flores *et al*., 2025).

## Supplementary data

The following supplementary data are available at *JXB* online.

Fig. S1. S-plot derived from the OPLS-DA model with a list of terpenes selected from the S-plot extremes.

Table S1. Fold change (FC) of selected terpenes in constitutive resin discriminating *Pinus pinaster* from *P. radiata*.

Table S2. Concentrations of the most significant terpenes affected by treatments.

Table S3. List of terpenes discriminating infected from wounded seedlings.

Table S4. List of terpenes discriminating by dpi.

Table S5. Genes involved in terpene biosynthesis.

## Abbreviations

DEG: differentially expressed gene
dpi: days post-inoculation
DRA: diterpene resin acid
DT: neutral diterpene
FC: fold change
I7, I26: *F. circinatum* isolates identified with numbers 7 and 26
MEP: methyl-erythritol phosphate
MI: mock inoculation with sterile water
MT: monoterpene
MVA: mevalonate
OPLS-DA: orthogonal partial least squares discriminant analysis
PCA: principal component analysis
PLS-DA: partial least squares discriminant analysis
PPC: pine pitch canker
ST: sesquiterpene
TRD: traumatic resin ducts
UW: unwounded (healthy) plants
VIP: variable importance in projection
