# Supplementary material for: Resin-based response of Pinus pinaster and P. radiata during infection by Fusarium circinatum
Source: J Exp Bot. 2026 Jan 20;77(8):2490–505. doi: 10.1093/jxb/erag023 (PMC13080375; doi:10.1093/jxb/erag023)
Supplement: erag023_Supplementary_Data [file erag023_supplementary_data.zip › jexbot315982-file002.pdf]

Table S1. Fold change (FC) of selected terpenes in constitutive resin discriminating *Pinus pinaster* from *P. radiata*

| *P. pinaster* (P) vs. *P. radiata* (R) | Group | FC (P/R) | 1/FC | $Log_2$ FC |
|---|---|---|---|---|
| phenylethyl butyrate | MT | 0.001 | 773.6 | -9.5954 |
| citronellal | MT | 0.002 | 635.5 | -9.3118 |
| citronellol | MT | 0.003 | 309.6 | -8.2743 |
| (E,E)-farnesol | ST | 0.008 | 133.2 | -7.0578 |
| piperitone | MT | 0.009 | 109.3 | -6.7725 |
| trans-piperitol | MT | 0.014 | 74.0 | -6.21 |
| bicyclogermacrene | ST | 0.017 | 60.3 | -5.9139 |
| phenylethyl propionate | MT | 0.017 | 57.5 | -5.8455 |
| thymol | MT | 0.028 | 35.9 | -5.1641 |
| cis-isopinocamphone | MT | 0.030 | 33.7 | -5.076 |
| trans-sabinene hydrate | MT | 0.036 | 27.6 | -4.7875 |
| farnesyl isovalerate | ST | 0.039 | 25.7 | -4.6824 |
| sabinene | MT | 0.044 | 22.7 | -4.5038 |
| (E)-citral | MT | 0.0 | und[a] | -4.1695 |
| unknown 10 | MT | 0.063 | 15.8 | -3.9778 |
| germacrene-D-4-ol | ST | 0.074 | 13.6 | -3.766 |
| 4-terpineol | MT | 0.075 | 13.3 | -3.7285 |
| trans caryophyllene | ST | 8.02 | | 3.0042 |
| isolongifolene | ST | 8.44 | | 3.0778 |
| cis-beta-farnesene | ST | 8.45 | | 3.0781 |
| copaborneol | ST | und | | 3.1351 |
| camphene | MT | 9.09 | | 3.1848 |
| alpha-ylangene | ST | und | | 3.1901 |
| neophytadiene | ST | und | | 3.2603 |
| cadina-1(6),4-diene | ST | und | | 3.3642 |
| sandaracopimaral | DT | 10.64 | | 3.4116 |

| | | | |
|---|---|---|---|
| germacrene D | ST | 10.94 | 3.4509 |
| pimaric acid | DRA | 12.15 | 3.6024 |
| alpha-amorphene | ST | 13.64 | 3.7696 |
| alpha-cadinol | ST | 13.65 | 3.7705 |
| alpha-longipinene | ST | und | 3.8435 |
| sandaracopimarinol | DT | 14.55 | 3.8633 |
| 15-isopimaradiene | DT | 14.60 | 3.8676 |
| labda-8(17),13E-dien-15-al | DT | 16.72 | 4.0637 |
| guaiol | ST | und | 4.4637 |
| unknown 30.7 | ST | 35.83 | 5.1631 |
| geranyl acetate | MT | 51.22 | 5.6785 |
| alpha-copaene | ST | 54.38 | 5.765 |
| alpha-cubebene | ST | 59.96 | 5.906 |
| cinnamyl valerate | MT | 60.69 | 5.9233 |
| alpha-muurolene | ST | 82.50 | 6.3664 |
| linalyl acetate | MT | 88.12 | 6.4613 |
| bornyl acetate | MT | 113.35 | 6.8247 |
| epibicyclosesquiphellandrene | ST | 114.85 | 6.8436 |
| valencene | ST | 1071.3 | 10.065 |

[a] und: undefined

Supplementary Table S2. Contents (µg $g^{-1}$ plant) and groups (G) of the 30 terpenes included in the heatmap built on abundances after treatments (*Fusarium circinatum* inoculation (I), mock inoculation (MI) and unwounded (UW)) applied to *Pinus pinaster* and *P. radiata* seedlings and measured at 12 and 19 dpi. Terpenes with a VIP score >1.3 were selected from the PLS-DA models (validation parameters: $R^2$= 0.9, $Q^2$= 0.75; p value=0.001 for *P. pinaster* and $R^2$= 0.85, $Q^2$=0.74; p value<0.0001 for *P. radiata*).

| *Pinus pinaster* | | | | | | | | | | | |
|---|---|---|---|---|---|---|---|---|---|---|---|
| | I12 | | I19 | | MI12 | | MI19 | | UW[a] | | G |
| | Avg[b] | St d[b] | Avg | St d | Avg | St d | Avg | St d | Avg | St d | |
| (e,e)-farnesol | 0.8 | 0.36 | 1.1 | 0.65 | 0.2 | 0.06 | 0.2 | 0.31 | 0.2 | 0.18 | ST |
| diterpenic alcohol | 4.3 | 1.82 | 6.0 | 2.36 | 3.5 | 2.46 | 0.3 | 0.32 | 1.3 | 1.51 | DT |
| labda-8(17),13-dien-15-al | 1.5 | 0.99 | 2.6 | 1.73 | 0.7 | 0.44 | 0.0 | 0.00 | 0.3 | 0.31 | DT |
| M+272 43.5 | 0.5 | 0.33 | 0.6 | 0.35 | 0.6 | 0.34 | 0.0 | 0.00 | 0.1 | 0.24 | DT |
| unknown15 | 0.3 | 0.44 | 0.7 | 1.22 | 0.1 | 0.03 | 0.0 | 0.00 | 0.0 | 0.02 | MT |
| 19-Nor-6,8,11,13-abietatetraene | 2.5 | 1.27 | 2.8 | 1.21 | 2.1 | 0.79 | 0.2 | 0.27 | 0.6 | 0.37 | DT |
| p-cymen-8-ol | 0.5 | 0.19 | 0.5 | 0.32 | 0.2 | 0.04 | 0.0 | 0.00 | 0.0 | 0.03 | MT |
| piperitone | 0.4 | 0.19 | 0.4 | 0.18 | 0.2 | 0.11 | 0.1 | 0.10 | 0.1 | 0.01 | MT |
| trans-piperitol | 0.3 | 0.12 | 0.2 | 0.11 | 0.1 | 0.00 | 0.0 | 0.00 | 0.1 | 0.00 | MT |
| 4-epidehydroabietol | 1.0 | 0.30 | 1.2 | 0.53 | 0.5 | 0.15 | 0.2 | 0.31 | 0.1 | 0.12 | DT |
| alpha-bisabolene | 0.2 | 0.20 | 0.1 | 0.06 | 0.0 | 0.01 | 0.0 | 0.00 | 0.0 | 0.01 | ST |

| | | | | | | | | | | | |
|---|---|---|---|---|---|---|---|---|---|---|---|
| 8,15-pimaradien-18-al | 0.6 | 0.30 | 0.8 | 0.45 | 0.4 | 0.05 | 0.0 | 0.00 | 0.0 | 0.03 | DT |
| 19-Nor-4,8,11,13-abietatetraene | 38.3 | 22.44 | 39.8 | 17.21 | 30.6 | 12.03 | 6.6 | 4.18 | 8.1 | 5.55 | DT |
| pinocarvone | 0.4 | 0.13 | 0.6 | 0.50 | 0.3 | 0.05 | 0.5 | 0.22 | 0.1 | 0.12 | MT |
| caryophyllene oxide | 5.1 | 1.21 | 6.9 | 2.86 | 3.5 | 0.09 | 4.8 | 2.41 | 0.6 | 1.64 | ST |
| dehydroabietal | 4.7 | 1.46 | 6.8 | 1.86 | 2.6 | 0.22 | 2.8 | 0.44 | 0.7 | 0.15 | DT |
| pinocamphone | 1.0 | 0.24 | 1.4 | 0.57 | 0.5 | 0.09 | 0.7 | 0.19 | 0.2 | 0.07 | MT |
| myrtenol | 5.0 | 1.75 | 6.3 | 4.84 | 1.9 | 0.46 | 3.4 | 1.07 | 0.4 | 0.44 | MT |
| cis-pinocarveol | 1.8 | 0.57 | 2.4 | 1.51 | 0.8 | 0.20 | 1.4 | 0.52 | 0.2 | 0.23 | MT |
| 8,11,13-abietatriene | 1.1 | 0.34 | 1.6 | 0.57 | 0.7 | 0.21 | 0.7 | 0.19 | 0.1 | 0.02 | DT |
| beta-pinone | 0.7 | 0.17 | 0.9 | 0.51 | 0.4 | 0.12 | 0.5 | 0.17 | 0.0 | 0.03 | MT |
| borneol | 10.6 | 3.86 | 12.4 | 6.12 | 3.4 | 0.52 | 5.4 | 1.75 | 0.5 | 0.87 | MT |
| cis-verbenol | 1.1 | 0.32 | 1.3 | 1.19 | 0.5 | 0.11 | 0.6 | 0.36 | 0.0 | 0.18 | MT |
| oplopane | 0.3 | 0.09 | 0.4 | 0.23 | 0.2 | 0.09 | 0.1 | 0.14 | 0.0 | 0.03 | ST |
| verbenone | 0.5 | 0.19 | 0.7 | 0.87 | 0.2 | 0.04 | 0.2 | 0.20 | 0.0 | 0.12 | MT |
| methyl-camphenilol | 4.5 | 1.31 | 6.7 | 2.41 | 2.9 | 0.50 | 1.7 | 0.26 | 0.8 | 0.17 | MT |
| cis-isopinocamphone | 0.7 | 0.34 | 1.1 | 1.15 | 0.1 | 0.04 | 0.2 | 0.31 | 0.0 | 0.19 | MT |
| manoyl oxide | 6.1 | 4.51 | 9.2 | 5.31 | 0.6 | 0.31 | 0.7 | 0.95 | 0.4 | 0.45 | DT |
| 13-epimanool | 2.7 | 1.59 | 3.6 | 2.05 | 0.6 | 0.46 | 0.3 | 0.28 | 0.2 | 0.12 | DT |
| 4-terpineol | 0.5 | 0.14 | 0.6 | 0.21 | 0.3 | 0.08 | 0.2 | 0.08 | 0.2 | 0.00 | MT |

| *Pinus radiata* | | | | | | | | | | | |
|---|---|---|---|---|---|---|---|---|---|---|---|
| | I12 | | I19 | | MI12 | | MI19 | | UW[a] | | G |
| | Avg[b] | St d[b] | Avg | St d | Avg | St d | Avg | St d | Avg | St d | |
| valencene | 0.0 | 0.00 | 16.6 | 40.35 | 0.0 | 0.01 | 0.0 | 0.00 | 0.0 | 0.01 | ST |
| cis-isopinocamphone | 3.3 | 2.38 | 15.7 | 10.32 | 1.1 | 0.26 | 1.9 | 0.29 | 1.0 | 0.24 | MT |
| 4-epidehydroabietol | 1.7 | 0.97 | 6.7 | 1.17 | 2.0 | 0.62 | 2.1 | 0.50 | 0.4 | 0.34 | DT |
| oxohydroxyresinic acid 51.9 | 47.3 | 22.23 | 140.8 | 64.81 | 48.2 | 11.46 | 51.6 | 30.23 | 15.3 | 21.61 | DRA |
| 4-hydroxy-18-nor-abietane | 3.1 | 0.81 | 7.0 | 2.53 | 2.1 | 0.40 | 2.7 | 0.46 | 0.6 | 0.86 | DT |
| dehydroabietal | 16.4 | 3.72 | 36.4 | 8.79 | 14.4 | 3.99 | 13.5 | 3.22 | 2.8 | 0.60 | DT |
| 8,11,13-abietatriene | 2.9 | 1.09 | 5.8 | 3.09 | 2.1 | 0.39 | 2.1 | 0.48 | 0.4 | 0.20 | DT |
| alpha-bisabolene | 2.5 | 1.83 | 1.4 | 0.86 | 0.3 | 0.07 | 0.3 | 0.06 | 0.1 | 0.09 | ST |
| abietal | 25.9 | 6.32 | 55.6 | 9.65 | 12.3 | 2.98 | 9.2 | 1.63 | 5.1 | 1.08 | DT |
| epi-manoyl oxide | 11.9 | 11.61 | 12.0 | 9.25 | 3.7 | 3.49 | 2.4 | 0.92 | 0.7 | 0.41 | DT |
| cis-verbenol | 0.8 | 0.47 | 4.5 | 2.59 | 0.5 | 0.14 | 0.8 | 0.17 | 0.2 | 0.01 | MT |
| cis-pinocarveol | 3.2 | 2.22 | 12.7 | 7.16 | 1.4 | 0.36 | 2.4 | 0.61 | 0.4 | 0.03 | MT |
| β-pinone | 1.4 | 0.57 | 6.0 | 3.87 | 1.0 | 0.36 | 1.5 | 0.32 | 0.2 | 0.01 | MT |
| p-cymene | 8.2 | 1.04 | 18.5 | 2.69 | 4.0 | 0.94 | 3.2 | 0.43 | 1.3 | 0.19 | MT |
| abietol | 9.8 | 1.89 | 14.5 | 3.76 | 5.7 | 1.32 | 3.6 | 1.36 | 1.1 | 1.51 | DT |

| | | | | | | | | | | | |
|---|---|---|---|---|---|---|---|---|---|---|---|
| p-cymen-8-ol | 4.7 | 1.18 | 10.3 | 2.70 | 2.0 | 0.39 | 2.0 | 0.87 | 0.3 | 0.02 | MT |
| sandaracopimarinol | 5.2 | 1.86 | 10.2 | 1.30 | 2.5 | 0.88 | 2.9 | 1.14 | 1.0 | 0.05 | DT |
| trans-4-thujanol | 0.8 | 0.28 | 1.6 | 0.42 | 0.5 | 0.06 | 0.6 | 0.21 | 0.2 | 0.07 | MT |
| (Z,E)-farnesyl isovalerate | 5.3 | 2.85 | 24.1 | 13.62 | 3.6 | 0.68 | 3.6 | 0.95 | 1.3 | 0.16 | ST |
| myrtenol | 8.9 | 3.98 | 38.7 | 22.39 | 3.5 | 0.93 | 4.7 | 1.33 | 1.1 | 0.34 | MT |
| sandaracopimaral | 3.8 | 1.10 | 9.1 | 1.72 | 2.3 | 0.75 | 2.3 | 0.73 | 1.0 | 0.10 | DT |
| oplopane | 1.8 | 1.94 | 5.5 | 1.17 | 3.3 | 0.92 | 4.2 | 2.47 | 0.2 | 0.33 | ST |
| 7-oxodehydroabietic acid | 1063 | 988.2 | 1403 | 802.81 | 2281 | 158.4 | 11.2 | 5.11 | 16.3 | 5.41 | DRA |
| 15- hydroxydehydroabietic acid | 112.8 | 90.86 | 163.8 | 63.69 | 260.8 | 60.97 | 9.2 | 2.72 | 8.1 | 5.85 | DRA |
| hydroxydehydroabietic acid 52.0 | 2270.4 | 1500.8 | 2841 | 1352.3 | 4290.8 | 227.93 | 303.9 | 122.48 | 42.3 | 16.05 | DRA |
| Seco I acid | 76.6 | 8.70 | 85.6 | 38.06 | 83.0 | 3.26 | 22.9 | 5.46 | 5.2 | 3.27 | DRA |
| Seco II acid | 69.5 | 6.24 | 75.1 | 35.78 | 71.5 | 7.08 | 21.7 | 4.81 | 4.4 | 0.53 | DRA |
| unknown15 | 1.7 | 0.78 | 3.4 | 1.75 | 1.6 | 0.22 | 0.9 | 0.60 | 0.2 | 0.02 | MT |
| alpha-cadinol | 1.9 | 0.47 | 2.6 | 1.23 | 2.2 | 0.76 | 1.8 | 0.65 | 0.3 | 0.38 | ST |
| resinic acid derivative 52.8 | 611.5 | 356.28 | 942.3 | 304.94 | 770.2 | 526.09 | 280.9 | 92.97 | 38.4 | 11.41 | DRA |
| pimaric acid | 825.6 | 120.50 | 1197.1 | 522.28 | 853.1 | 47.21 | 595.8 | 212.62 | 103.4 | 3.17 | DRA |

[a] Pooled data from 12 and 19 dpi

[b] Mean and standard deviation

Seco= 1,3-dimethyl-2-ethyl-(3-isopropylphenyl)-cyclohexencarboxilic

Supplementary Table S3. Fold change (FC) of terpenes discriminating *F. circinatum*-infected from wounded seedlings of *Pinus pinaster* and *P. radiata* at 12 and 19 dpi.

| | Infected (I) *vs.* wounding (MI) | Group | FC (I/MI[a]) | $log_2$ FC |
|---|---|---|---|---|
| *P. pinaster* 12 dpi | manoyl oxide | DT | 12.3 | 3.616 |
| | cis-isopinocamphone | MT | 10.7 | 3.417 |
| | resinic 49.0 | DRA | 9.2 | 3.208 |
| | 15-hydroxydehydroabietic | DRA | 8.7 | 3.120 |
| | abieta-8(14),13(15)-diene | DT | 7.7 | 2.945 |
| | bicyclogermacrene | ST | 7.6 | 2.930 |
| | Seco I | DRA | 7.5 | 2.911 |
| | 7-oxodehydroabietic | DRA | 6.6 | 2.715 |
| | 13(16),14-labdien-8-ol | DT | 6.4 | 2.688 |
| *P. pinaster* 19 dpi | p-cymen-8-ol | MT | 5.2 | 2.388 |
| | manoyl oxide | DT | 4.9 | 2.306 |
| | diterpenic alcohol | DT | 4.7 | 2.220 |
| | pimarol | DT | 4.5 | 2.185 |
| | abietol | DT | 4.0 | 1.999 |
| | labda-8(17),13E-dien-15-al | DT | 3.1 | 1.631 |
| | 8,15-pimaradien-18-al | DT | 2.3 | 1.193 |
| | isolongifolene | ST | 2.1 | 1.087 |
| *P. radiata* 12 dpi | resinic 49.4 | DRA | 59.0 | 5.884 |
| | 13-epimanool | DT | 11.8 | 3.560 |
| | hydroxyabietic 51.8 | DRA | 9.7 | 3.284 |
| | 8,15-pimaradien-18-al | DT | 0.16 | -2.606 |
| | beta-elemene | ST | 0.16 | -2.647 |
| | labda-8(17),13E-dien-15-al | DT | 0.14 | -2.851 |
| | 15-isopimaradiene | DT | 0.13 | -2.963 |
| | abieta-8(14),13(15)-diene | DT | 0.12 | -3.031 |
| | beta-cubebene | ST | 0.12 | -3.052 |
| | alpha-copaene | ST | 0.11 | -3.121 |
| | pimarol | DT | 0.11 | -3.164 |
| | alpha-amorphene | ST | 0.09 | -3.497 |
| | alpha-cubebene | ST | 0.02 | -5.670 |
| *P. radiata* 19 dpi | 7-oxodehydroabietic | DRA | 125.1 | 6.967 |
| | 15-hydroxydehydroabietic | DRA | 17.8 | 4.155 |
| | hydroxydehydroabietic 52.0 | DRA | 9.3 | 3.225 |
| | myrtenol | MT | 8.2 | 3.041 |
| | cis-isopinocamphone | MT | 8.0 | 3.008 |
| | alpha-terpinene | MT | 7.2 | 2.854 |
| | alpha-cubebene | ST | 6.8 | 2.773 |
| | abietal | DT | 6.0 | 2.593 |
| | p-cymene | MT | 5.7 | 2.511 |

Terpene identification was done from the S-plot derived from the respective OPLS-DA model. Validation parameters for the OPLS-DA models were: $R^2Y=0.946$ and $Q^2=0.901$ with p-value=0.014 (*P. pinaster* at 12 dpi); $R^2Y=0.906$ (p-value=0.028) and $Q^2=0.843$ (p-value=0.015) (*P. pinaster* at 19 dpi); $R^2Y=0.932$ (p-value=0.083) and $Q^2=0.870$ (p-value=0.026) (*P. radiata* at 12 dpi); and $R^2Y=0.910$ (p-value=0.007) and $Q^2=0.868$ (p-value=0.007) (*P. radiata* at 19 dpi). Only those terpenes with $log_2$fold change (FC)>|2| and significant ($P<0.05$) separation of

classes were selected. Seco= 1,3-dimethyl-2-ethyl-(3-isopropylphenyl)-cyclohexencarboxilic. Colored terpenes are common in both species and included in Table 1: light green is for those with increased content in infected *P. pinaster*, and decreased in *P. radiata*; light orange, when content increased in both species but at different dpi.

Supplementary Table S4. Fold change (FC) of terpenes discriminating classes of *Pinus pinaster* infected seedlings at 12 dpi from 19 dpi, and *P. radiata* at 12 dpi from 19 dpi.

| | 12 dpi (I12) *vs*. 19 dpi (I19) | Group | FC (I12/I19) | $Log_2$ FC |
|---|---|---|---|---|
| *P. pinaster* | citronellic acid | MT | 38.4 | 5.263 |
| | 7-oxodehydroabietic | DRA | 10.7 | 3.426 |
| | abieta-8(14),13(15)-diene | DT | 8.7 | 3.124 |
| | resinic 47.7 | DRA | 0.13 | -2.923 |
| | resinic 46.9 | DRA | 0.09 | -3.552 |
| | farnesyl isovalerate | ST | 0.04 | -4.707 |
| | alpha-terpineol | MT | 0.02 | -5.981 |
| | resinic 49.4 | DRA | 0.01 | -6.557 |
| *P. radiata* | trans-sabinene hydrate | MT | 0.18 | -2.506 |
| | labda-8(17),13E-dien-15-al | DT | 0.15 | -2.727 |
| | isolongifolene | ST | 0.14 | -2.855 |
| | alpha-cubebene | ST | 0.13 | -2.969 |
| | beta-cubebene | ST | 0.12 | -3.027 |
| | 15-isopimaradiene | DT | 0.11 | -3.132 |
| | 8,15-pimaradien-18-al | DT | 0.11 | -3.178 |
| | alpha-amorphene | ST | 0.10 | -3.288 |
| | verbenone | MT | 0.10 | -3.394 |
| | alpha-cadinene | ST | 0.09 | -3.457 |
| | pimarol | DT | 0.08 | -3.560 |
| | delta-cadinene | ST | 0.08 | -3.613 |
| | alpha-copaene | ST | 0.08 | -3.689 |
| | beta-elemene | ST | 0.05 | -4.300 |
| | beta-gurjunene | ST | 0.03 | -5.073 |

Terpene identification was done from the S-plot derived from the OPLS-DA model. Validation parameters for OPLS-DA models: $R^2Y=0.839$, p-value=0.016 and $Q^2=0.715$ with p-value=0.002 for *P. pinaster*; and $R^2Y=0.969$, p-value=0.002 and $Q^2=0.905$, p-value=0.002 for *P. radiata*. Only those terpenes with $log_2$fold change (FC)>|2|and significant (P<0.05) separation of classes were selected.

Supplementary Table S5. Differentially expressed genes of the corresponding enzymes related to plant terpene biosynthesis identified from the transcriptome of *Pinus pinaster* under *Fusarium circinatum* infection at 3, 5 and 10 days postinoculation (dpi)

| Pathway map in KEGG | GenBank ID | Enzyme | Abbrv. | Log2 (Fold change) value | | |
|---|---|---|---|---|---|---|
| | | | | 3 dpi | 5 dpi | 10 dpi |
| | Backbone biosynthesis: MVA Pathway | | | | | |
| map00900 / M00095 | GIBR01016634.1 | acetyl-CoA C-acetyltransferase | AACT | 6.37 | 9.32 | 11.75 |
| map00900 / M00095 | GIBR01011662.1 | acetyl-CoA C-acetyltransferase | AACT | 0.55 | 1.79 | 2.14 |
| map00900 / M00095 | GIBR01019493.1 | acetyl-CoA C-acetyltransferase | AACT | 0.00 | 1.03 | 0.00 |
| map00900 / M00095 | GIBR01023709.1 | hydroxymethylglutaryl-CoA synthase | HMGS | 6.41 | 9.25 | 12.91 |
| map00900 / M00095 | GIBR01020327.1 | hydroxymethylglutaryl-CoA synthase | HMGS | 1.04 | 3.06 | 3.20 |
| map00900 / M00095 | GIBR01003908.1 | hydroxymethylglutaryl-CoA reductase (NADPH) | HMGR | 3.03 | 5.75 | 7.34 |
| map00900 / M00095 | GIBR01003731.1 | hydroxymethylglutaryl-CoA reductase (NADPH) | HMGR | 0.00 | 0.00 | 2.19 |
| map00900 / M00095 | GIBR01003526.1 | hydroxymethylglutaryl-CoA reductase (NADPH) | HMGR | 0.00 | 0.00 | -1.47 |
| map00900 / M00095 | GIBR01003609.1 | hydroxymethylglutaryl-CoA reductase (NADPH) | HMGR | 0.00 | 0.00 | -0.55 |
| map00900 / M00095 | GIBR01000845.1 | hydroxymethylglutaryl-CoA reductase (NADPH) | HMGR | 0.00 | 0.00 | -1.41 |
| map00900 / M00095 | GIBR01012984.1 | hydroxymethylglutaryl-CoA reductase (NADPH) | HMGR | 0.00 | 0.00 | 2.20 |
| map00900 / M00095 | GIBR01024060.1 | hydroxymethylglutaryl-CoA reductase (NADPH) | HMGR | 0.00 | 0.00 | 1.19 |
| map00900 / M00095 | GIBR01014570.1 | hydroxymethylglutaryl-CoA reductase (NADPH) | HMGR | 6.12 | 9.77 | 9.98 |
| map00900 / M00095 | GIBR01011283.1 | mevalonate kinase | MVK | 0.57 | 1.73 | 2.65 |

| | | | | | | |
|---|---|---|---|---|---|---|
| map00900 / M00095 | GIBR01020600.1 | Phosphomevalonate kinase | PMVK | 0.00 | 0.73 | 1.55 |
| map00900 / M00095 | GIBR01007153.1 | diphosphomevalonate decarboxylase | MVD | 0.90 | 2.35 | 2.95 |
| map00900 / M00095 | GIBR01010326.1 | isopentenyl-diphosphate delta-isomerase | IPPI | 0.00 | 1.33 | 2.42 |
| map00900 / M00095 | GIBR01004097.1 | farnesyl diphosphate synthase | FPS | 5.69 | 7.87 | 11.53 |
| map00900 / M00095 | GIBR01017274.1 | farnesyl diphosphate synthase | FPS | 6.02 | 8.42 | 13.71 |
| | Backbone biosynthesis: MEP pathway | | | | | |
| map00900 / M00096 | GIBR01004877.1 | 1-deoxy-D-xylulose-5-phosphate synthase | DXS | 0.00 | 0.00 | -0.79 |
| map00900 / M00096 | GIBR01023989.1 | 1-deoxy-D-xylulose-5-phosphate synthase | DXS | 0.00 | -0.85 | -0.64 |
| map00900 / M00096 | GIBR01006179.1 | 1-deoxy-D-xylulose 5-phosphate reductoisomerase | DXR | 0.00 | 0.00 | -1.49 |
| map00900 / M00096 | GIBR01004606.1 | 2-C-methyl-D-erythritol 4-phosphate cytidylyltransferase | MCT | 0.00 | 0.00 | -0.55 |
| map00900 / M00096 | GIBR01001644.1 | 4-(cytidine 5'-diphospho)-2-C-methyl-D-erythritol kinase | CMK | 0.00 | 0.00 | -0.70 |
| map00900 / M00096 | GIBR01019111.1 | (E)-4-hydroxy-3-methylbut-2-enyl-diphosphate synthase (ferredoxin) | HDS | 0.00 | 0.00 | -1.13 |
| map00900 / M00096 | GIBR01015821.1 | 4-hydroxy-3-methylbut-2-en-1-yl diphosphate reductase | HDR | 0.00 | 0.00 | -1.46 |
| map00900 / M00096 | GIBR01008025.1 | 4-hydroxy-3-methylbut-2-en-1-yl diphosphate reductase | HDR | 0.00 | 0.00 | -1.27 |
| map00900 / M00096 | GIBR01011521.1 | geranyl-diphosphate synthase | GPPS | 0.00 | -0.95 | -2.81 |
| map00900 / M00096 | GIBR01016240.1 | geranyl-diphosphate synthase | GPPS | 0.00 | 0.00 | 0.88 |
| map00900 / M00096 | GIBR01022823.1 | geranyl-diphosphate synthase | GPPS | 0.00 | 0.00 | -0.83 |
| map00900 / M00096 | GIBR01010692.1 | geranylgeranyl diphosphate synthase | GGPPS | 0.00 | -0.63 | -1.53 |
| map00900 / M00096 | GIBR01010947.1 | geranylgeranyl diphosphate synthase | GGPPS | 0.00 | 0.00 | -3.14 |

| | | | | | | |
|---|---|---|---|---|---|---|
| map00900 / M00096 | GIBR01018086.1 | geranylgeranyl diphosphate synthase | GGPPS | 0.00 | 0.00 | -1.07 |
| | Monoterpene biosynthesis | | | | | |
| map00902 | GIBR01002598.1 | alpha-pinene synthase | | 0.00 | 1.06 | 3.99 |
| map00902 | GIBR01014919.1 | alpha-pinene synthase | | 0.00 | 0.00 | -2.62 |
| map00902 | GIBR01009062.1 | alpha-pinene synthase | | 0.00 | 0.00 | -0.82 |
| map00902 | GIBR01013221.1 | alpha-pinene synthase | | 0.00 | 0.00 | -1.58 |
| map00902 | GIBR01006549.1 | alpha-pinene synthase | | 0.00 | 0.00 | -2.24 |
| map00902 | GIBR01019315.1 | 2-methyl-3-buten-2-ol synthase | | 0.00 | -0.67 | -1.95 |
| map00902 | GIBR01013541.1 | (+)-3-carene synthase | | 0.00 | 0.00 | -1.80 |
| map00902 | GIBR01003339.1 | myrcene synthase | | 0.00 | 0.00 | -2.00 |
| map00902 | GIBR01012308.1 | (4S)-limonene synthase | | 0.00 | 0.00 | -0.70 |
| | Diterpene biosynthesis | | | | | |
| map00904 | GIBR01004577.1 | Copalyl diphosphate synthase | | 0.00 | 0.00 | -2.54 |
| map00904 | GIBR01014597.1 | Pimaradiene synthase | | 1.33 | 2.25 | 7.31 |
| map00904 | GIBR01016591.1 | Ent-kaurene synthase | | 0.00 | -0.57 | -1.53 |
| map00904 | GIBR01010635.1 | isopimara-7,15-diene synthase | | 0.00 | 0.00 | -1.84 |
| map00904 | GIBR01013923.1 | isopimara-7,15-diene synthase | | 0.00 | 0.00 | 2.92 |
| map00904 | GIBR01007972.1 | levopimaradiene synthase | | 0.00 | 0.00 | -2.53 |
| map00904 | GIBR01020605.1 | levopimaradiene synthase | | 0.00 | 0.00 | -1.29 |

| | | | | | |
|---|---|---|---|---|---|
| map00904 | GIBR01002780.1 | isopimaradiene synthase | 0.00 | 0.00 | -1.14 |
| map00904 | GIBR01010761.1 | abietadienol abietadienal | 0.00 | -0.90 | -3.02 |
| | Sesquiterpene biosynthesis | | | | |
| map00909 | GIBR01003798.1 | Alpha-farnesene synthase | 6.26 | 8.02 | 8.99 |
| map00909 | GIBR01014133.1 | Alpha-farnesene synthase | 5.91 | 7.02 | 7.22 |
| map00909 | GIBR01018017.1 | Alpha-farnesene synthase | 1.29 | 1.26 | 2.13 |
| map00909 | GIBR01015392.1 | Alpha-bisabolene synthase | 3.64 | 4.22 | 3.68 |
| map00909 | GIBR01018349.1 | Alpha-longifolene synthase | 0.00 | 0.00 | -3.28 |
| map00909 | GIBR01012270.1 | Alpha-longipinene synthase | 4.46 | 6.62 | 6.02 |
| map00909 | GIBR01002641.1 | Delta-selinene synthase | 0.00 | 0.00 | -1.67 |
| map00909 | GIBR01003339.1 | 1(10),5-germacradien-4-ol synthase | 0.00 | 0.00 | -2.00 |
