# Supplementary material for: Resin-based response of Pinus pinaster and P. radiata during infection by Fusarium circinatum
Source: J Exp Bot. 2026 Jan 20;77(8):2490–505. doi: 10.1093/jxb/erag023 (PMC13080375; doi:10.1093/jxb/erag023)
Supplement: erag023_Supplementary_Data [file jexbot315982-file001.pdf › jexbot315982-file001.pdf]

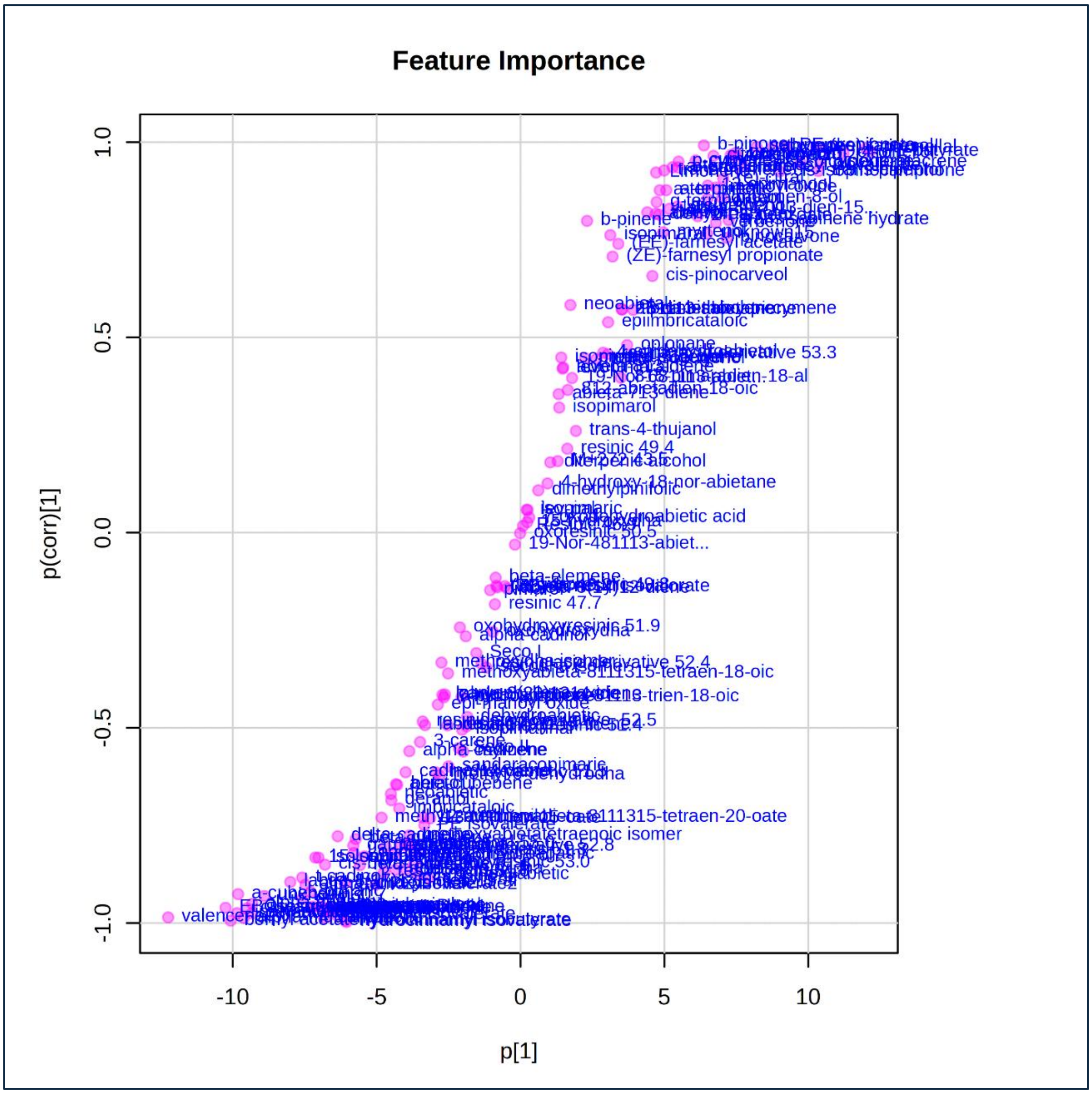

Fig. S1. S-plot derived from the OPLS-DA model with a list of terpenes selected from the S-plot extremes. The x-axis is the P loading profile, and the y-axis is the P correlation loading vector of the predictive component of the OPLS-DA model of constitutive resin of *P. pinaster* (P) *vs. P. radiata* (R). Validation parameters for the model were: $R^2Y=0.999$ and $Q^2=0.989$ with p-value=0.002. Only those terpenes with log_2fold change (FC)>|2| and significant ($P<0.05$) separation of classes were.
